# Hepatocytes reprogram liver macrophages involving control of TGF-**β** activation, influencing liver regeneration and injury

**DOI:** 10.1097/HC9.0000000000000208

**Published:** 2023-07-24

**Authors:** Stephanie D. Wolf, Christian Ehlting, Sophia Müller-Dott, Gereon Poschmann, Patrick Petzsch, Tobias Lautwein, Sai Wang, Barbara Helm, Marcel Schilling, Julio Saez-Rodriguez, Mihael Vucur, Kai Stühler, Karl Köhrer, Frank Tacke, Steven Dooley, Ursula Klingmüller, Tom Luedde, Johannes G. Bode

**Affiliations:** 1 Department of Gastroenterology, Hepatology and Infectious Disease, Faculty of Medicine & Düsseldorf University Hospital, Heinrich Heine University, Düsseldorf, Germany; 2 Institute for Computational Biomedicine, Faculty of Medicine & Heidelberg University Hospital, Heidelberg University, Heidelberg, Germany; 3 Molecular Proteomics Laboratory, BMFZ, Heinrich Heine University Düsseldorf, Düsseldorf, Germany; 4 Genomics & Transcriptomics Laboratory, BMFZ, Heinrich Heine University, Düsseldorf, Germany; 5 Molecular Hepatology Section, Medical Faculty Mannheim, Heidelberg University, Mannheim, Germany; 6 Division of Systems Biology of Signal Transduction, German Cancer Research Center (DKFZ), DKFZ-ZMBH Alliance, Heidelberg, Germany; 7 Institute of Molecular Medicine, Proteome Research, Medical Faculty and University Hospital, Heinrich-Heine-University Düsseldorf, Düsseldorf, Germany; 8 Department of Hepatology & Gastroenterology, Charité Universitätsmedizin Berlin, Campus Virchow-Klinikum (CVK) and Campus Charité Mitte (CCM), Berlin, Germany

## Abstract

Macrophages play an important role in maintaining liver homeostasis and regeneration. However, it is not clear to what extent the different macrophage populations of the liver differ in terms of their activation state and which other liver cell populations may play a role in regulating the same.

**Methods:**

Reverse transcription PCR, flow cytometry, transcriptome, proteome, secretome, single cell analysis, and immunohistochemical methods were used to study changes in gene expression as well as the activation state of macrophages in vitro and in vivo under homeostatic conditions and after partial hepatectomy.

**Results:**

We show that F4/80^+^/CD11b^hi^/CD14^hi^ macrophages of the liver are recruited in a C-C motif chemokine receptor (CCR2)–dependent manner and exhibit an activation state that differs substantially from that of the other liver macrophage populations, which can be distinguished on the basis of CD11b and CD14 expressions. Thereby, primary hepatocytes are capable of creating an environment *in vitro* that elicits the same specific activation state in bone marrow–derived macrophages as observed in F4/80^+^/CD11b^hi^/CD14^hi^ liver macrophages *in vivo*. Subsequent analyses, including studies in mice with a myeloid cell–specific deletion of the TGF-β type II receptor, suggest that the availability of activated TGF-β and its downregulation by a hepatocyte-conditioned milieu are critical. Reduction of TGF-βRII-mediated signal transduction in myeloid cells leads to upregulation of IL-6, IL-10, and SIGLEC1 expression, a hallmark of the activation state of F4/80^+^/CD11b^hi^/CD14^hi^ macrophages, and enhances liver regeneration.

**Conclusions:**

The availability of activated TGF-β determines the activation state of specific macrophage populations in the liver, and the observed rapid transient activation of TGF-β may represent an important regulatory mechanism in the early phase of liver regeneration in this context.

## Graphical abstract

**Figure:**
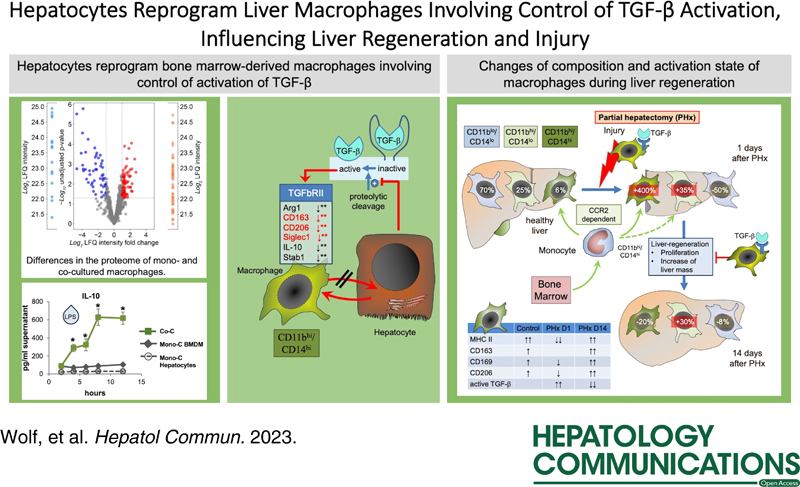

## INTRODUCTION

Liver macrophages are heterogeneous. Among them, tissue-resident macrophages that colonize the liver in the fetal period are capable of self-renewal. They can be distinguished from macrophages that originate from bone marrow–derived monocytes and migrate to the liver in postfetal stages.^1^ The latter is characterized, among others, by high expression of the integrin alpha-M beta-2 subunit CD11b^1^ and also CD14, a subunit of the LPS receptor complex and a characteristic marker of bone marrow–derived monocyte or macrophage populations in humans,^2^ but also in other mammals, including mice.^3,4^ Here, C-C motif chemokine receptor (CCR2)^5^ is critical for their recruitment, especially under inflammatory conditions. Beyond this, the different macrophage populations in the liver perform different tasks or functions. For example, tissue-resident liver macrophages are thought to exert predominantly homeostatic functions, whereas recruited macrophages appear to be rather involved in the context of inflammatory responses.^1^ However, the assignment of individual macrophage populations to different tasks and functions is partially fuzzy and not yet well understood. The question of how clearly the boundaries between different macrophage populations can be drawn here is still open. At the very least, recent work based on lineage tracing experiments suggests that recruited macrophages can adapt their phenotype to that of tissue-resident macrophages of the liver under the influence of the liver microenvironment.^6^ The classification is even more complex since macrophages are characterized by a high plasticity that allows them to adapt their physiology to the surrounding microenvironment. The changes in their activation state are reflected in an altered expression of various genes and proteins, such as the scavenger receptors CD163 or CD206, and adhesion molecules, such as SIGLEC1, but also other factors including cytokines, such as IL-6 or IL-10,^7,8^ and metabolic enzymes, such as arginase 1 (Arg1). To account for the wide range of activation states that macrophages can adopt, the originally used nomenclature distinguishing between classically activated macrophages (M1) and alternatively activated or wound-healing macrophages (M2) was replaced by a nomenclature based on the stimulus that triggers a particular activation state under standardized experimental conditions.^9^

To date, it is largely unclear to what extent tissue-resident and recruited macrophage populations of the liver differ in their activation state under homeostatic conditions. Likewise, it is not yet sufficiently clear to what extent this changes under pathological conditions and which intercellular communication signals are involved. In this context, the influence of hepatocytes on the activation state of macrophages is of particular interest, as they are the main source of so-called acute-phase proteins.^10^ The composition of these proteins exerts an enormous modifying influence on the microenvironment prevailing in the liver sinusoid under both homeostatic and pathological conditions. Thus, for example, they act as binding proteins and, thereby, inactivate or stabilize cytokines or as protease inhibitors and decisively influence the degradation or cleavage of other molecules.^10^ This is fostered by the particular anatomy of sinusoids with large fenestrated endothelia allowing free exchange of soluble molecules. In this way, hepatocyte-derived factors easily enter the intraluminal milieu of the sinusoids, where liver macrophages are mainly localized.

In general, macrophages exert a Janus-faced role in tissues, as they play a central role in both the induction of tissue damage and the orchestration of repair and regeneration processes. This is also true for the liver, where, in the case of regeneration, they are central sources of cytokines such as TNF-α and IL-6-like cytokines. Together with growth factors, these cytokines crucially regulate the initial, proliferation, and termination phases of liver regeneration.^11^ Here, IL-6-type cytokines mediate protective anti-inflammatory effects^12^ and directly promote local proliferation and restoration of tissue-resident macrophages.^13^ Accordingly, a reduction in the number and functionality of liver macrophages leads to a significant impairment of liver regeneration and subsequent recovery of homeostasis.^14,15^ However, to what extent and with what dynamics the composition or activation states of the different macrophage populations in the remnant liver change during the regeneration process, for example, after a single damaging event, such as partial hepatectomy (PHx), are unclear.

Here, we investigate the impact of hepatocytes on the activation state of recruited macrophages, identify relevant signals under homeostatic conditions, and investigate their relevance for liver regeneration after PHx.

## Methods

### Animals

Animal housing and all the experimental procedures were reviewed and approved by the North Rhine-Westphalian State Agency for Nature, Environment, and Consumer Protection under the reference numbers 81-02.04.2018.A149, 81-02.04.2017.A406, 81-02.04.2013.A464 and 81-02.04.2010.A279 and performed in accordance with the Animal Research: Reporting of In Vivo Experiments guidelines. Mice were housed in cages under standard laboratory conditions (22 -24°C temperature, 55% -60% relative humidity, and 12 h light/dark cycle), with standard food and water provided ad libitum. The hygiene monitoring took place quarterly according to the recommendations of the Federation of European Laboratory Animal Science Associations for the health monitoring of laboratory animal facilities. The hygiene status of the animal husbandry was specific pathogen-free (SPF). For the experiments C57BL/6J mice, B6.129P2-Lyz2tm1(cre)Ifo/J (LysM-cre) mice paired with B6;129-Tgfbr2tm1Karl/J (TGF-βRII-flox), and B6.129S4-Ccr2tm1 (Ccr2^−/−^) mice (male animals, 8- to 12-week old) were used.

### Coculture model

A detailed description is provided in the Supplemental Materials including Supplemental Figure S1, http://links.lww.com/HC9/A410. In brief, highly purified primary murine hepatocytes were prepared, and 0.8 Mio per well were embedded in a collagen matrix. Bone marrow–derived macrophages (BMDMs) were splitted on day 7 of differentiation, and 0.1 Mio cells were seeded per transwell. BMDMs were allowed to settle for one day and thereafter transferred to 6-well plates containing hepatocytes or to respective control wells.

### Analysis of proteome and secretome

Hepatocytes, BMDM, and culture supernatants from monocultures and cocultures were processed and prepared for mass spectrometric analysis. As described in detail in the Supplemental Materials, subsequently, peptides were separated using liquid chromatography and mass spectrometric analysis.

### Transcriptome analysis

The total RNA was prepared and quantified, and quality was determined. Synthesis of cDNA and subsequent biotin labeling of cDNA was performed and hybridized to Applied Biosystems Clariom S Mouse Gene Expression Microarrays, stained by streptavidin/phycoerythrin conjugate, and scanned. The whole procedure and the subsequent data analysis are described in detail in the Supplemental Materials.

### Single-cell analysis

Nonparenchymal liver cells (NPLCs) were isolated with a Liver Dissociation Kit. Death cells were excluded by Zombie. After Fc block cells were labeled with antibodies against CD45 and F4/80. Vital CD45^+^/F4/80^+^ cells were subsequently sorted by FACS. Single-cell processing was carried out on the 10X Chromium Controller system and sequenced on the NextSeq-550 system with a mean sequencing depth of ~50,000 reads/cell. The entire experimental procedure and the processing of the data obtained are described in detail in the Supplemental Materials, http://links.lww.com/HC9/A410.

### Additional methods

Due to limited space, the methods for isolation of NPLC and the analysis by flow cytometry, as well as the methods for RNA isolation, cDNA synthesis, real-time PCR, analysis of cytokine production, immunohistochemistry, the performance of the 2/3 partial hepatectomy, and serum biochemistry, are described in the Supplemental Materials, used antibodies and primers are listed in the Supplemental Tables S3-S5, http://links.lww.com/HC9/A410

### Statistical analysis

Statistics were calculated using GraphPad Prism 6 software. Results were analyzed using the nonparametric 2-tailed Mann-Whitney *U* test as indicated in the figure legends; *p* ≤ 0.05 was considered significant. For a more detailed description, see Supplemental Materials, http://links.lww.com/HC9/A410

### Data availability statement

The single-cell RNA sequencing data for this project were deposited on the European Bioinformatics Insititute (EBI) database under accession number E-MTAB-11727 (https://www.ebi.ac.uk/arrayexpress/experiments/E-MTAB-11727) and the proteome data under the accession number PXD012136 (https://www.ebi.ac.uk/pride/archive/projects/PXD012136). Raw data from the RNA Microarray were deposited on GEO under accession number GSE201467 (https://www.ncbi.nlm.nih.gov/geo/query/acc.cgi?acc=GSE201467).

## Results

### CCR2-dependent recruited liver macrophages exhibit a unique activation state

**FIGURE 1:**
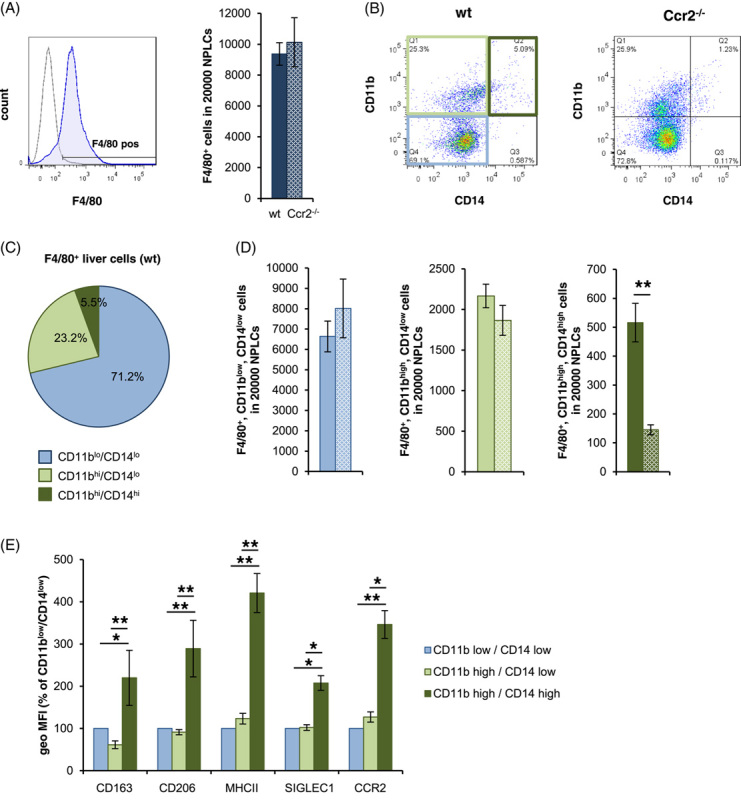
CCR2 dependently recruited liver macrophages are characterized by high expression of CD11b and CD14 and a distinctive pattern of activation markers. NPLCs were isolated from wt (filled bars) and for A, B, and D from CCR2^−/−^ (spotted bars) mice and analyzed by flow cytometry using antibodies against (A) F4/80 (blue) versus FMO control (gray). For (B–D), analysis was performed using antibodies against F4/80, CD11b, and CD14 and grouped on the basis of the expression intensity of CD11b and CD14 into F4/80^+^/CD11b^lo^/CD14^lo^ (bright blue), F4/80^+^/CD11b^hi^/CD14^lo^ (bright green), and F4/80^+^/CD11b^hi^/CD14^hi^ (dark green). (E) Analysis of the different subgroups characterized in B using additional antibodies against CD163, CD206, MHCII, SIGLEC1, and CCR2. The geometric MFIs of CD11b^hi/^CD14^hi^ and CD11b^hi/^CD14^lo^ cells are shown in relation to the respective CD11b^lo^/CD14^lo^ cells. The data are presented as means ± SEM (n = 6/genotype). Mann-Whitney *U* test, * for *p* ≤ 0.05 and ** for *p* ≤ 0.01. Abbreviations: CCR2, C-C motif chemokine receptor; NPLCs, nonparenchymal liver cells.

To characterize macrophages recruited to the liver, NPLCs from wild type and Ccr2^−/−^ mice were analyzed by flow cytometry using F4/80 as a macrophage marker (Figure 1A, gating strategy in Supplemental Figure S2, http://links.lww.com/HC9/A410). Based on the expression levels of the cell surface proteins CD11b and CD14, the F4/80^+^ cells were assigned to 3 different groups (Figure 1B). Of these, ~71% exhibited low surface expression of both CD11b and CD14 (F4/80^+^/CD11b^lo^/CD14^lo^), whereas 23% can be characterized as F4/80^+^/CD11b^hi^/CD14^lo^. Only a small proportion of cells (6%) exhibited a marker profile with high surface expression of CD11b and CD14 (F4/80^+^/CD11b^hi^/CD14^hi^) (Figure 1C), corresponding to the profile of BMDMs (Supplemental Figure S1D, http://links.lww.com/HC9/A410). In contrast to the other macrophage populations, the F4/80^+^/CD11b^hi^/CD14^hi^ population is substantially diminished in the liver tissue of CCR2-deficient mice (Figure 1B, D). This suggests that these macrophages are recruited in a CCR2-dependent manner and can be distinguished from the other populations by evidence of increased surface expression of CD14. Interestingly, the activation state of this macrophage population fundamentally differs from the other 2 populations and is characterized by enhanced surface expression of MHCII, CD163, CD206, SIGLEC1, and CCR2 (Figure 1E).

### Hepatocytes generate a micromilieu modifying the activation state of monocyte-derived macrophages

As leading producers of acute-phase proteins, hepatocytes are important determinants of the environment in the liver sinusoid.^10^ To interrogate the interaction between hepatocytes and recruited macrophages, a coculture model was established using highly purified hepatocytes embedded in a collagen sandwich to prevent their epithelial-to-mesenchymal transition (Supplemental Figure S1, http://links.lww.com/HC9/A410). As a model for recruited macrophages, BMDMs were used in accordance with the consensus recommendations for the study of the activation state of macrophages.^9^

**FIGURE 2:**
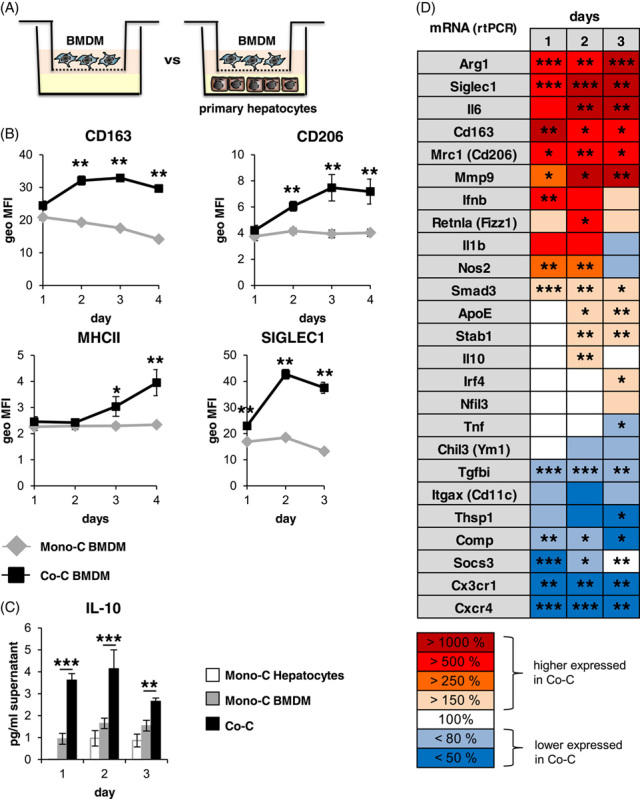
Hepatocytes induce a particular activation state in BMDM. (A) Scheme of the coculture system used. (B) Monocultivated and cocultivated BMDM (n = 5–6) were analyzed for CD163, CD206, MHCII, or SIGLEC1 expression using flow cytometry and (C) for IL-10 release using ELISA (n = 7–8). (D) Heat map of mRNA expression levels of different genes in monocultivated or cocultivated BMDM after 1–3 days of (co)cultivation (Supplemental Figure S3, http://links.lww.com/HC9/A410). Data are presented as means ± SEM. Mann-Whitney *U* test, * for *p* ≤ 0.05, ** for *p* ≤ 0.01, and *** for *p* ≤ 0.001. Abbreviations: BMDM, bone marrow–derived macrophages.

Our results demonstrated that hepatocytes induce a distinct activation state in BMDM characterized by the upregulation of the surface markers CD163, CD206, MHCII, and SIGLEC1 and increased the release of IL-10 (Figure 2A–C). Moreover, transcript levels of Arg1, Siglec1, Il6, Il10, and Mmp9 were significantly upregulated in BMDM in the presence of hepatocytes, whereas the transcripts of Tgfbi, Thsp1, and Cxcr4 were downregulated (Figure 2D, Supplemental Figure S3, http://links.lww.com/HC9/A410).

These hepatocyte-imprinted changes in the activation state of BMDM (H-BMDM) are accompanied by significant changes in the H-BMDM response to inflammatory stimuli, such as Lipopolysaccharide (LPS), characterized by an enhanced induction of mRNAs for genes including Il10, Arg1, Nos2, and Ifnb1 and a faster downregulation of Il6 and Tnf (Supplemental Figure S4, http://links.lww.com/HC9/A410). Consistent with these observations, there was a significantly greater LPS-induced increase in the concentration of IL-10, IFN-β, and, at the early time points, a significantly accelerated increase in IL-6 protein levels under coculture conditions compared to monocultured BMDM, while the concentration of TNF-α was decreased in the later time points in the presence of hepatocytes (Supplemental Figure S5, http://links.lww.com/HC9/A410). In the supernatant of monocultured hepatocytes, the concentrations of IL-10, IFN-β, IL-6, and TNF-α were either close to the detection limit (IL-10) or in the range of monocultured BMDM (IFN-β) or below the respective concentrations of monocultivated and cocultivated BMDM (TNF-α and IL-6). This indicates that the observed changes were due to the modifying influence of hepatocytes on BMDM and not to altered synthesis in the hepatocyte (Supplemental Figure S5, http://links.lww.com/HC9/A410).

### Cocultivation changes the proteome and transcriptome of macrophages, but not of hepatocytes, and reduces the availability of activated TGF-β

**FIGURE 3:**
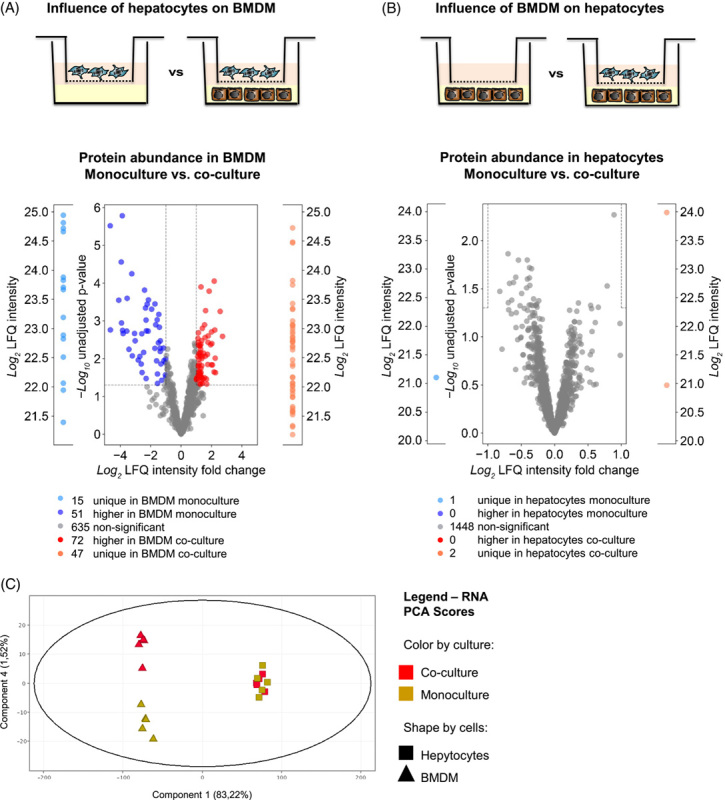
Hepatocytes induce a marked change in cocultivated BMDM, but BMDM has only a minor influence on cocultured hepatocytes. Proteome (mass spectrometry, A and B) and transcriptome (microarray, C) were analyzed in monocultivated and cocultivated macrophages and/or hepatocytes. (A) Volcano plots comparing the proteome of monocultivated versus cocultivated macrophages and (B) monocultivated versus cocultivated hepatocytes. In both Volcano plots, log10-transformed *p* values are plotted against log-transformed fold change in abundance between monoculture and coculture. The nonaxial horizontal line denotes *p* = 0.05 (significance threshold), and the vertical lines indicate fold change = 2 (threshold cutoff). All unique proteins are sorted according to their log2 LFQ intensities. (C) PCA scores of transcriptome data show the respective overall normalized mRNA expression for monocultivated (red) and cocultivated (yellow) hepatocytes (■) and BMDM (▲). In each case, 5 independent samples were analyzed. Abbreviations: BMDM, bone marrow–derived macrophages; LFQ, label-free quantification; PCA, principal component analysis.

To assess the reciprocal influence of intercellular communication between hepatocytes and monocyte-derived macrophages, the global proteome of primary murine hepatocytes and BMDM in monoculture or coculture was analyzed by mass spectrometry (Figure 3A, B). The statistical analysis of the proteome data (Supplemental Table S1, http://links.lww.com/HC9/A410, S2, http://links.lww.com/HC9/A410) revealed that hepatocytes have a major influence on BMDM (Figure 3A), whereas surprisingly BMDMs have rather little effect on hepatocytes (Figure 3B). Consistently, the transcriptome of BMDM, but not of hepatocytes, is significantly influenced by the culture conditions (Figure 3C). These results suggest that, under homeostatic conditions, hepatocytes release factors that significantly affect gene and protein expression in macrophages, but not *vice versa*. However, this unidirectional communication in coculture changes to bidirectional after stimulation with LPS. Here, a strong increase in transcript expression was observed in hepatocytes, including acute-phase genes and genes for intercellular communication signals, such as chemokines (Supplemental Figure S6, http://links.lww.com/HC9/A410).

**FIGURE 4:**
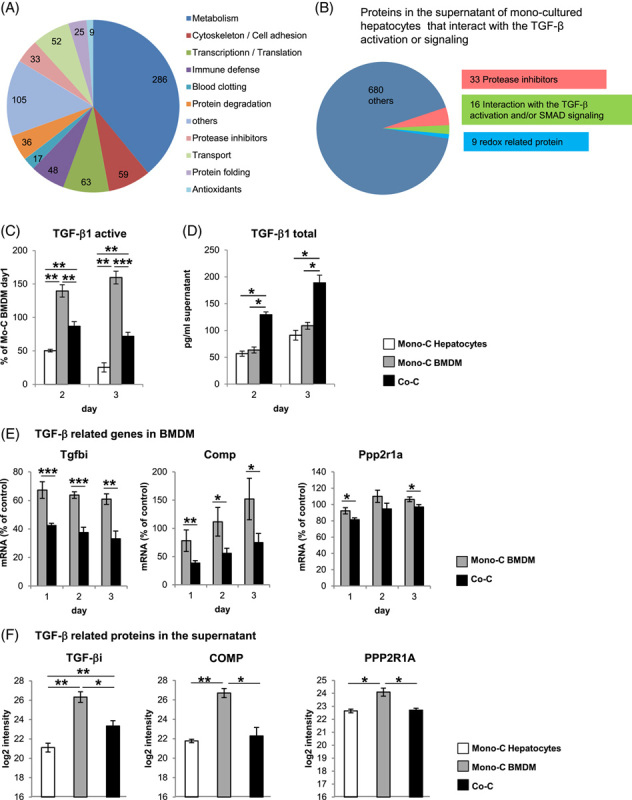
Hepatocytes reduce the concentration of active TGF-β1 in the supernatant of cocultivated BMDM. (A) Pie chart of all proteins detected by mass spectrometry in the supernatant of monocultivated hepatocytes grouped based on protein function and (B) those proteins that could modulate TGF-β activation and/or signaling. (C and D) Supernatants of monocultivated or cocultivated BMDM and/or hepatocytes were collected after 2 and 3 days of culture and analyzed for active (after concentration, C) or total (D) TGF-β1 using ELISA. Data of active TGF-β1 are expressed in relation to monocultivated BMDM at day 1 (n = 4–8). (E) Differences in the mRNA expression of Tgfbi, Comp, and Ppp2r1a in monocultivated and cocultivated BMDM after 1–3 days of cultivation (n = 5–7) and (F) in the concentration of these factors in the supernatant determined by mass spectrometry after 2 days of cultivation (n = 5). Data are presented as means ± SEM. Mann-Whitney *U* test, * for *p* ≤ 0.05, ** for *p* ≤ 0.01, and *** for *p* ≤ 0.001. Abbreviations: BMDM, bone marrow–derived macrophages.

The selected coculture model allows intercellular communication only through soluble molecules, suggesting that the influence of hepatocytes on the activation state of BMDM is mediated through factors secreted by hepatocytes. Therefore, the secretome of monocultured primary hepatocytes was analyzed by mass spectrometry. In these analyses, ~700 proteins were detected, mainly belonging to the metabolic category (Figure 4A). In addition, at least 33 protease inhibitors are secreted by hepatocytes, all of which could potentially interfere with proteolytic activation of factors such as TGF-β, as has been shown, for example, for α_2_M.^10,16^ Furthermore, 16 factors have been identified, which could also affect TGF-β activation (Figure 4B, Supplemental Figure S7A, B, http://links.lww.com/HC9/A410), for example, by binding and stabilizing the latent form of TGF- β, as has been shown for the matrix protein ECM1.^10,17,18^ These data suggest that control of TGF-β-activation may represent a possible mechanism by which hepatocytes influence the activation state of BMDM. Consistent with this assumption, the amount of active TGF-β in supernatants of cocultures is effectively suppressed compared with monocultured BMDM although the total amount of TGF-β approximately doubles (Figure 4C, D). The observed levels of activated TGF-β were reflected by decreased expression of TGF-β-regulated genes and proteins, such as TGF-β-induced protein (Tgfbi), oligomeric cartilage matrix protein (Comp), and protein phosphatase 2 scaffold subunit 1 (Ppp2r1) in cocultured H-BMDM compared with monocultured BMDM (Figure 4E, F).

### Disrupted myeloid cell–specific TGF-β signal transduction influences the activation state of macrophages *in vitro* and *in vivo*

**FIGURE 5:**
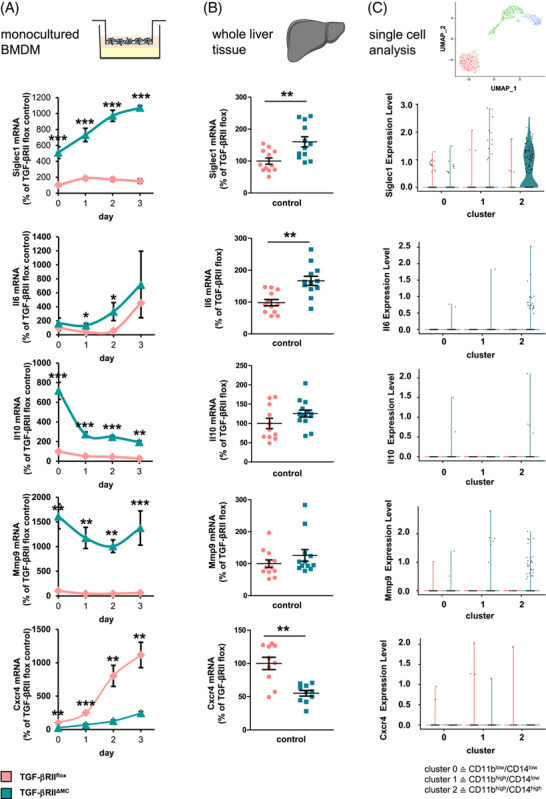
Impairment of TGF-βRII signaling in myeloid cells modulates gene expression of genes in BMDM, liver tissue, and liver macrophages. Expression of Siglec1, Il6, Il10, Mmp9, and Cxcr4 was analyzed by reverse transcription PCR in total mRNA isolated from (A) BMDM (n = 5–8) or (B) whole liver tissue (n = 9–12) derived from TGF-βRII^flox^ or TGF-βRII^ΔMC^ mice. Data are expressed as means ± SEM. Mann-Whitney *U* test, * for *p* ≤ 0.05, ** for *p* ≤ 0.01, and *** for *p* ≤ 0.001. (C) Cluster-dependent analysis of the expression of these genes in liver macrophage subpopulations of TGF-βRII^flox^ or TGF-βRII^ΔMC^ mice discriminated on the basis of single-cell RNA analysis (n = 2). Abbreviations: PHx, partial hepatectomy; NPLCs, nonparenchymal liver cells.

Based on the above observations, we investigated whether impairment of TGF-β signaling affects the activation state of macrophages. For this purpose, mice with a myeloid cell–specific deletion of TGF-βRII (TGF-βRII^ΔMC^) were generated. Accordingly, surface expression of TGF-βRII was significantly reduced in BMDMs generated from these animals (Supplemental Figure S8A, http://links.lww.com/HC9/A410). Remarkably, impairment of TGF-βRII-mediated signal transduction in monocultured TGF-βRII^ΔMC^ BMDM mimicked the effect of hepatocytes on the activation state of H-BMDM, upregulated the expression of transcripts encoding Siglec1, Il6, Il10, and Mmp9, and downregulated the expression of Cxcr4 (Figure 5A). This observation was also reflected *in vivo* in the whole liver tissue (Figure 5B), suggesting that the deletion of TGF-βRII does not exclusively affect the activation state of recruited macrophages. Accordingly, BMDM lacking TGF-βRII or treated with neutralizing TGF-β antibodies showed an increased expression of CD163 and CD206, whereas stimulation with TGF-β1 reduced them (Supplemental Figure S8B–D, http://links.lww.com/HC9/A410).

To characterize the impact of disrupted TGF-β-dependent signal transduction on the activation state of the different macrophage populations *in vivo*, CD45^+^/F4/80^+^ cell populations from the liver of control mice (TGF-βRII^flox^) and TGF-βRII^ΔMC^ mice were subjected to single-cell RNA sequencing analysis. In these data sets, we identified macrophage populations based on the expression of typical macrophage marker genes, such as Clec4f, Adgre1 (F4/80), and Cd68 (Supplemental Figure S9, http://links.lww.com/HC9/A410), and divided them into three subpopulations based on their gene expression pattern (clusters 0, 1, and 2) (Supplemental Figure S10A, http://links.lww.com/HC9/A410). Cluster 2 of these cells showed the highest percentage of CD11b and CD14 expressing cells, and cluster 0 showed the lowest (Supplemental Figure S10B, C, http://links.lww.com/HC9/A410). The percentages of the cells in these clusters were in striking agreement with the percentages obtained for the three populations defined by flow cytometry (Supplemental Figure S10D, http://links.lww.com/HC9/A410). In line with the results illustrated in Figure 5A, B, the comparative analysis of the three clusters indicated that transcripts such as Siglec1, Il6, Il10, and Mmp9 are increased on abrogation of TGF-βRII signaling, whereas the expression of Cxcr4 is decreased (Figure 5C). Overall, the CD11b^hi^/CD14^hi^ macrophages grouped in cluster 2 comprised the largest number of genes whose regulation was affected by deletion of TGF-βRII (Supplemental Figure S10E, http://links.lww.com/HC9/A410). This suggests that this macrophage group is most strongly influenced by signals mediated through TGF-βRII. However, this is not exclusively the case, as the absence of TGF-βRII also affects the expression of genes such as IL-6 or IL-10 in the other 2 clusters (Figure 5C).

In summary, these data suggest that, in the liver under homeostatic conditions, it is mainly the activation state of recruited F4/80^+^/CD11b^hi^/CD14^hi^ macrophages that are affected by TGF-β, and TGF-β does not affect all macrophage populations equally.

### Liver regeneration after PHx results in a CCR2-dependent rapid increase of F4/80^+^/CD11b^hi^/CD14^hi^ macrophages

To investigate changes in the composition of the different macrophage subpopulations in the liver after tissue injury, the PHx model was chosen to induce a single, sterile injury event. In this model, already, at day 2 after PHx, transaminases were almost normalized (Supplemental Figure S11A, http://links.lww.com/HC9/A410).

**FIGURE 6:**
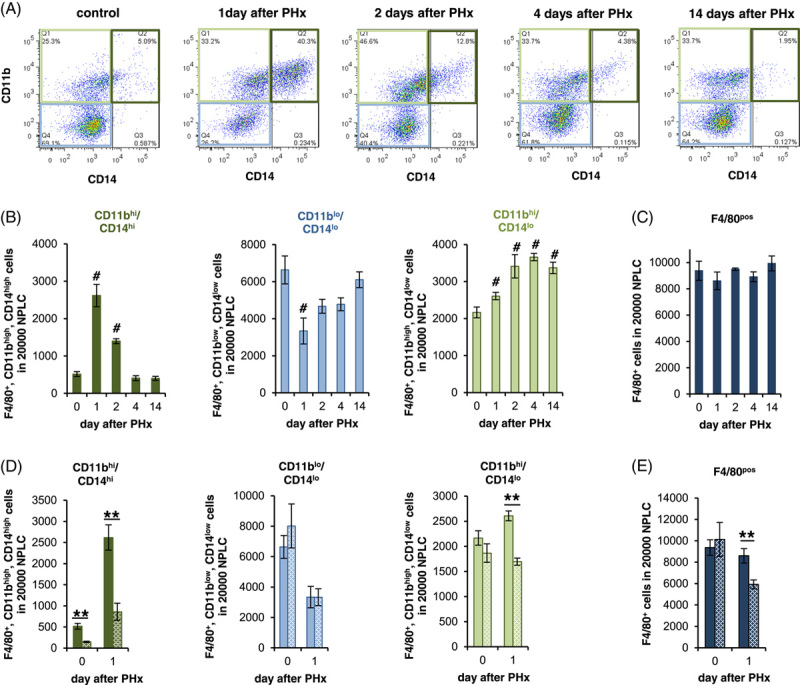
Recruitment of CD11b^hi^/CD14^hi^ macrophages during the course of liver regeneration is CCR2 dependent. PHx was performed in (A–E) wt (filled bars) or (D, E) CCR2^−/−^ (spotted bars) mice and animals were sacrificed after the time points indicated. (A) Gating strategy for subdividing F4/80-positive NPLCs based on their CD11b and CD14 expressions by flow cytometry. (B, D) Quantification of F4/80^+^/CD11b^hi^/CD14^hi^ (dark green), F4/80^+^/CD11b^lo^/CD14^lo^ (bright blue), and F4/80^+^/CD11b^hi^/CD14^lo^ (bright green) in 20,000 NPLC each and (C, E) sum of the F4/80^+^ macrophage subpopulations (dark blue). The data are presented as means ± SEM (n = 5–7). Significant differences to 0 h are indicated by # for *p* ≤ 0.05. Significant differences between wt and Ccr2^−/−^ animals were indicated by ** for *p* ≤ 0.01 (Mann-Whitney *U* test). Abbreviations: CCR2, C-C motif chemokine receptor; NPLCs, nonparenchymal liver cells; PHx, partial hepatectomy.

After PHx, there was initially a significant and rapid increase in F4/80^+^/CD11b^hi^/CD14^hi^ macrophages, which was transient and reached its maximum on the first day. At this stage, these cells accounted for one-third of all liver macrophages. Concurrently, the population of F4/80^+^/CD11b^lo^/CD14^lo^ macrophages decreased from 75% to about one-third. With a time delay, the F4/80^+^/CD11b^hi^/CD14^lo^ macrophage population increased and reached its maximum of about one-third of all liver macrophages around day 2 and then entered a plateau phase that lasted throughout the observation period until day 14. This increase was counterbalanced by a concomitant decrease in F4/80^+^/CD11b^hi^/CD14^hi^ macrophages, which returned to baseline levels not later than by day 4 (Figure 6A, B). Of note, these shifts in the composition of the different macrophage populations proceeded without significant changes in the total number of macrophages in liver tissue as indicated by histological (Supplemental Figure S12B, http://links.lww.com/HC9/A410) and flowcytometric measurements (Figure 6C).

Importantly, both the presence under homeostatic conditions and the increase in the F4/80^+^/CD11b^hi^/CD14^hi^ macrophage population at day 1 after PHx are CCR2-dependent (Figure 6D, Supplemental Figure S13, http://links.lww.com/HC9/A410), whereas deletion of CCR2 has no effect on the F4/80^+^/CD11b^lo^/CD14^lo^ population (Figure 6D). The observed decrease in the total population of F4/80^+^ macrophages observed at day 1 after PHx compared with wt animals in CCR2^−/−^ animals (Figure 6E) is most likely due to the lack of increase in the F4/80^+^/CD11b^hi^/CD14^lo^ population (Figure 6D).

### Transient activation of TGF-β during the early phase of liver regeneration coincides with substantial changes in the activation state of recruited macrophages

**FIGURE 7:**
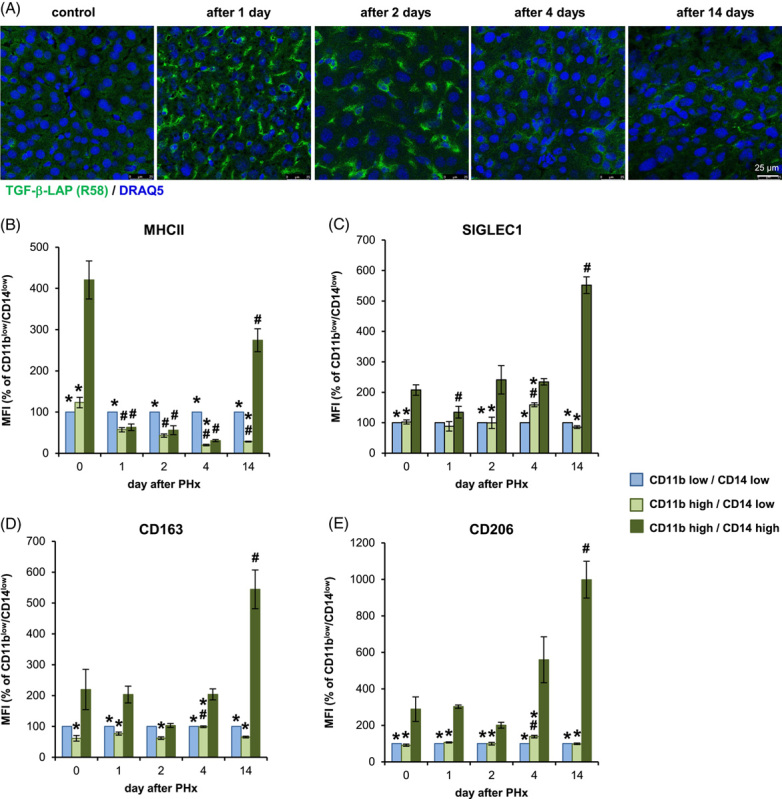
After PHx, TGF-β is strongly activated and may potentially affect the polarization of the F4/80^+^/CD11b^hi^/CD14^hi^ macrophage subpopulation. PHx was performed in wt mice, and animals were sacrificed after the time points indicated. (A) Sections of snap‐frozen liver tissue were stained with anti-TGF-β-LAP (green) and DRAQ5 (blue). Representative sections are shown (×25 magnification, scale bars: 25 µm). NPLCs were analyzed for expression of MHCII (B), SIGLEC1 (C), CD163 (D), and CD206 (E) in the three macrophage subpopulations distinguished by flow cytometry (like in Figures 1 and 6). The geometric MFI of CD11b^hi^/CD14^hi^ and CD11b^hi^/CD14^lo^ cells is shown relative to the respective CD11b^lo^/CD14^lo^ cells. Data are presented as means ± SEM (n = 5–6). Significant differences to the respective control at day 0 are indicated by # for *p* ≤ 0.05, and significant differences to the CD11b^hi^/CD14^hi^ population at the different time points are indicated by * for *p* ≤ 0.05 (Mann-Whitney *U* test). Abbreviations: NPLC, nonparenchymal liver cells; PHx, partial hepatectomy.

As an indicator of the activation of TGF-β the occurrence of the latency-associated peptide, the cleavage product that remains after the activation of latent TGF-β can be monitored.^17^ The course of detectability of latency-associated peptide by immunofluorescence analysis indicates that there is rapid and transient activation of TGF-β in the course of liver regeneration after PHx. TGF-β activation reached its maximum at day 1 after PHx and dropped significantly already at day 4 to return to baseline at day 14 at the latest (Figure 7A, Supplemental Figure S11B, http://links.lww.com/HC9/A410). This course of TGF-β activation correlates negatively with the increased surface expression of the markers MHCII, SIGLEC1, CD163, and CD206 by, in particular, F4/80^+^/CD11b^hi^/CD14^hi^ macrophages (Figure 7B–E), which becomes particularly evident in the later stages of regeneration. Remarkably, these changes were not detectable to a comparable extent in the other 2 macrophage populations, suggesting that the course of TGF-β activation and, in particular, the activation state of F4/80^+^/CD11b^hi^/CD14^hi^ macrophages are functionally linked.

### Loss of TGF-βRII-mediated signaling in myeloid cells reduces surgery-induced liver injury and improves liver regeneration after partial hepatectomy

**FIGURE 8:**
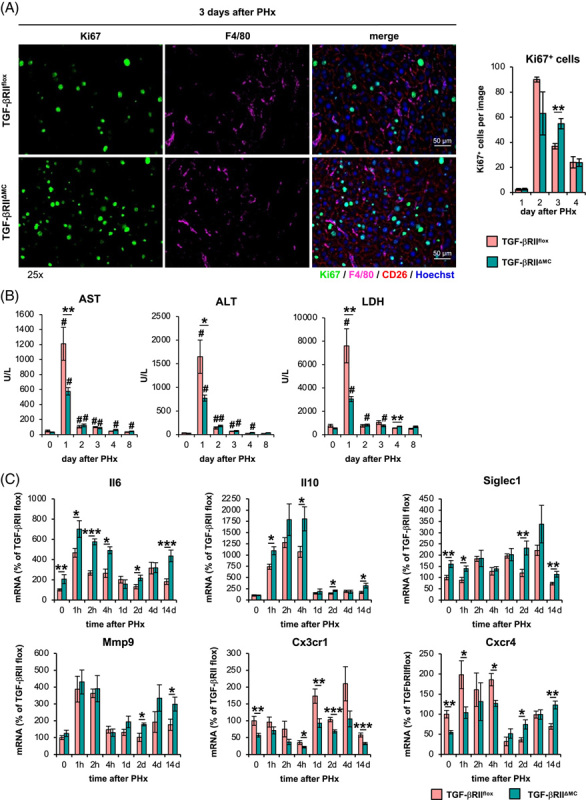
Impaired TGF-βRII signaling in myeloid cells prolongs hepatocyte proliferation and reduces injury after PHx. PHx was performed in TGF-βRII^flox^ or TGF-βRII^ΔMC^ mice, and animals were sacrificed after the time points indicated. (A) Sections of snap‐frozen liver tissue were analyzed using confocal microscopy after staining with antibodies specific for Ki67 (green), F4/80 (pink), CD26 (red), and Hoechst (blue). Representative sections are shown (×25 magnification, scale bars: 50 µm). Quantification of proliferating cell nuclei at day 1–4 after PHx (n = 3–5). (B) AST, ALT, and LDH activities in the serum of respective animals (n = 4–7). (C) Total mRNA was extracted from whole liver tissue, and transcript abundance of Il10, Il6, Siglec1, Mmp9, Cx3cr1, and Cxcr4 was determined by reverse transcription PCR (n = 5–10). The data are presented as means ± SEM. Significant differences to control (0) are indicated by # for *p* ≤ 0.05, and differences between TGF-βRII^ΔMC^ and TGF-βRII^flox^ mice are indicated by * for *p* ≤ 0.05, ** for *p* ≤ 0.01, and *** for *p* ≤ 0.001 (Mann-Whitney *U* test). Abbreviations: ALT, alanine aminotransferase; AST, aspartate aminotransferase; LDH, lactate dehydrogenase; PHx, partial hepatectomy.

Disruption of TGF-βRII-mediated signaling in myeloid cell populations resulted in a prolonged proliferation phase (Figure 8A Supplemental Figure S12A, http://links.lww.com/HC9/A410), which was associated with a markedly accelerated increase in liver weight in TGF-βRII^ΔMC^ mice on day 3 after PHx (Supplemental Figure S12D, http://links.lww.com/HC9/A410). These changes in TGF-βRII^ΔMC^ mice concomitantly led to a significant reduction in postoperative serum levels of ALT, AST, and LDH on day 1 after PHx (Figure 8B, Figure S14, http://links.lww.com/HC9/A410) compared respective controls.

Strikingly, both basal Il6 expression in liver tissue and the increase in the early phase between one and 4 hours after PHx were significantly enhanced in TGF-βRII^ΔMC^ mice compared with control animals (Figure 8C). These initial differences in Il6 expression between TGF-βRII^ΔMC^ and control animals disappeared as liver regeneration progressed but became prominent again in the late phase of liver regeneration at day 14. This observation is interesting because a series of works suggests that IL-6 mediates not only proregenerative^19^ but also protective effects in the context of liver regeneration.^12,20^ Thus, in the context of liver regeneration, IL-6 protects against the otherwise deleterious influence of LPS-triggered inflammatory responses^12^ and is able to compensate for the disruption of regeneration caused by the deletion of CD169-positive cells.^20^ Of note, apart from Il6, other indicator genes, such as Il10, Siglec1, Mmp9, Cx3cr1, and Cxcr4, whose expression is most markedly regulated in macrophages grouped in cluster 2, have their expression either enhanced or inhibited by TGF-βRII-mediated signaling during the course of liver regeneration (Figure 8C).

## DISCUSSION

In mammals, the liver harbors the largest pool of sessile macrophages in the organism. Consistently, under homeostatic conditions, >70% sessile macrophages (CD11b^lo^/CD14^lo^) constitute the majority of the macrophage populations in the liver (Figure 1C), whereas monocyte-derived macrophage populations recruited to the liver in a CCR2-dependent manner constitute a minority of less than 6%. This latter CCR2-dependent macrophage population is clearly identifiable by the high surface expression of the marker molecules CD11b and CD14, whereas the CCR2-independent macrophage populations account for >90% and are characterized overall by low surface expression of CD14 (Figure 1).

The data presented provide evidence that the activation state of CD11b^hi^/CD14^hi^ macrophages substantially differs from the other macrophage populations of the liver (Figures 1, 5–7). Thus, the environment prevailing in the liver sinusoid affects the activation state of individual macrophage populations differently, depending, among other things, on their origin. Thereby, the results of coculture experiments suggest that hepatocytes play an important role in this context by creating an environment suitable to induce an activation state in BMDM similar to that of CD11b^hi^/CD14^hi^ liver macrophages (Figures 2, 3, Supplemental Figure S3, http://links.lww.com/HC9/A410, S4, http://links.lww.com/HC9/A410). The data presented further suggest that active TGF-β is an environmental parameter by which this specific activation state is codetermined. Consistent with this, reduction of TGF-β signaling in myeloid cells by deletion of TGF-βRII in BMDM results in an activation state that phenocopies that of H-BMDM *in vitro* and that of CD11b^hi^/CD14^hi^ liver macrophages or the macrophage populations grouped under cluster 2 in the single-cell RNA analysis *in vivo* (Figure 5). However, the results of single-cell RNA analysis also suggest that, although the reduction in TGF-βRII signaling most strongly affects gene expression in macrophages of cluster 2, this is not exclusive to this group but also leads to changes in the other 2 macrophage populations grouped in clusters 0 and 1.

The availability of active TGF-β, in addition to regulation of the synthesis and release of latent TGF-β, is controlled in particular by the proteolytic cleavage of latent TGF-β into the latency-associated peptide and active TGF-β.^17^ The activation of TGF-β can be influenced by factors such as ECM1,^18^ α_2_M,^16^ AHSG,^21^ and Bikunin,^22^ which either stabilize the latent form or act as protease inhibitors preventing its cleavage. These factors are mainly produced by hepatocytes^10,16,18,21,22^ and were detected in the secretome of hepatocytes *in vitro* (Figure 4, Supplemental Figure S7B, http://links.lww.com/HC9/A410). In line with this assumption, preliminary results suggest that ECM1 at least has a tendency to simulate aspects of the influence of hepatocytes on BMDM (Supplemental Figure S15, http://links.lww.com/HC9/A410). However, ECM1, but also TGF-β, should be considered only as parts of a much more complex network that likely involves synergistic effects through which factors secreted by hepatocytes influence the activation state of liver macrophages. In this context, it should also be considered that hepatocytes themselves may be involved in controlling the availability of active TGF-β under certain circumstances since at least *in vitro* observations suggest that activation of latent TGF-β1 complexes can occur intracellularly in regenerating but not in normal hepatocytes.^23^

In summary, the data presented here suggest that inhibition of TGF-β activation in an environment created by coculture with hepatocytes or prevalent in the liver sinusoid contributes to the induction of a particular activation state in certain macrophage populations. Consistent with this, activation of TGF-β in the early phase of liver regeneration correlates with a loss of these unique activation features on CCR2-dependently recruited macrophages, and the decay of TGF-β activation in the late phase of liver regeneration is accompanied by a recovery of this particular activation state of CD11b^hi^/CD14^hi^ macrophages (Figure 7).

The data further suggest that the influence of TGF-β on the activation state of macrophages and, thus, on their function has an impact on the course of liver regeneration. Thereby, the reduction of signal transduction through the TGF-βRΙΙ in myeloid cells is particularly associated with a prolongation of the phase of cell division (Figure 8A) and a correspondingly faster increase in liver weight (Supplemental Figure S12D, http://links.lww.com/HC9/A410) and with an attenuation of the liver damage caused by the surgical intervention (Figure 8B). In this context,the blockade of this signal transduction particularly influences events that take place in the early phase of liver regeneration, such as the expression of Il6, the expression of which is significantly enhanced in liver tissue under both homeostatic and in the course of the first hours after PHx. This increase in expression of Il6 was mainly but not exclusively observable in the macrophage population corresponding to cluster 2 of the single-cell analysis and coincides with damage reduction and extension of the proliferation phase of hepatocytes, both of which are known to be supported by IL-6.^12,19^ In this context, it is interesting that CD11b^hi^/CD14^hi^ macrophages are distinguished from other macrophages by high expression of SIGLEC1, as work by others has demonstrated that depletion of SIGLEC1-positive cells significantly impairs liver regeneration after PHx. An effect that has also been attributed to decreased IL-6 production after the depletion of SIGLEC1-positive cells.^20^

The observation reported here that signaling through the TGF-βRII particularly affects the early phase of liver regeneration is consistent with the observations of others in which either deletion of components of intracellular TGF signaling, such as beta2SP,^24^ or disruption of signaling of TGF-β in hepatocytes had a beneficial effect on liver regeneration.^25^

In summary, our data support the notion that inhibition of TGF-β signal transduction in the early phase of PHx or even before PHx may be an approach to improve liver regeneration and patient outcome after PHx. An important aspect of the effect of TGF-β is that it influences the activation state of macrophages in the liver and, through this, the inflammatory response that is involved in the orchestration of the early phase of liver regeneration. However, it is also quite conceivable that this influence of activated TGF-β on the activation state of macrophage populations of the liver, and the resulting changes in the inflammatory milieu are also relevant for the development of fibrosis and cancer in the context of chronic liver diseases, as these situations may concur with over activation of the TGF-β pathway.

## Supplementary Material

SUPPLEMENTARY MATERIAL
