# Supplementary material for: Hepatocytes reprogram liver macrophages involving control of TGF-β activation, influencing liver regeneration and injury
Source: Hepatol Commun. 2023 Jul 24;7(8):e0208. doi: 10.1097/HC9.0000000000000208 (PMC10368377; doi:10.1097/HC9.0000000000000208)
Supplement: SUPPLEMENTARY MATERIAL [file hc9-7-e0208-s001.pdf]

Supplemental Materials

Hepatocytes reprogram liver macrophages involving control of TGF-β activation, influencing liver regeneration and injury

Stephanie D. Wolf[1], Christian Ehlting[1], Sophia Müller-Dott[2], Gereon Poschmann[3], Patrick Petzsch[4], Tobias Lautwein[4], Sai Wang[5], Barbara Helm[6], Marcel Schilling[6], Julio Saez-Rodriguez[2], Mihael Vucur[1], Kai Stühler[3,7], Karl Köhrer[4], Frank Tacke[8], Steven Dooley[5], Ursula Klingmüller[6], Tom Luedde[1], Johannes G. Bode[1*]

[1] Department of Gastroenterology, Hepatology and Infectious disease, Faculty of Medicine & Düsseldorf University Hospital, Heinrich-Heine-University, Düsseldorf, Germany

[2] Institute for Computational Biomedicine, Heidelberg University, Faculty of Medicine & Heidelberg University Hospital, Rupprecht Karls University, Heidelberg, Germany

[3] Molecular Proteomics Laboratory, BMFZ, Heinrich Heine University Düsseldorf, Düsseldorf, Germany

[4] Genomics & Transcriptomics Laboratory, BMFZ, Heinrich Heine University, Düsseldorf, Germany

[5] Molecular Hepatology Section, Medical Faculty Mannheim, Heidelberg University, Mannheim, Germany

[6] Division of Systems Biology of Signal Transduction, German Cancer Research Center (DKFZ), DKFZ-ZMBH Alliance, Heidelberg, Germany

[7] Institute of Molecular Medicine, Proteome Research, Medical Faculty and University Hospital, Heinrich-Heine-University Düsseldorf, Düsseldorf, Germany

[8] Department of Hepatology & Gastroenterology, Charité Universitätsmedizin Berlin, Campus Virchow-Klinikum (CVK) and Campus Charité Mitte (CCM), Berlin, Germany

Table of contents:

Supplemental Materials & Methods 3
Fig. S1 13
Fig. S2 15
Fig. S3 17
Fig. S4 19
Fig. S5 20
Fig. S6 21
Fig. S7 22
Fig. S8 23
Fig. S9 25
Fig. S10 26
Fig. S11 27
Fig. S12 29
Fig. S13 30
Fig. S14 31
Fig. S15 32
Table S1 33
Table S2 34
Table S3 35
Table S5 37
References specific for supplementary materials 38

Supplemental Materials & Methods

Animals – C57BL/6J wt mice from Janvier, $CCR2^{-/-}$ and TGF-βRII-loxP mice from Jackson, breed with LysM-cre mice on a C57BL/6 background, were used for the experiments. The TGF-βRII-loxP mice were generated and described by Levéen *et al.* 2002 [1], the $CCR^{-/-}$ mice by Boring *et al.* 1997 [2]. Animal housing and all the experimental procedures were reviewed and approved by the North Rhine-Westphalian State Agency for Nature, Environment, and Consumer Protection (LANUV) under the reference numbers 81-02.04.2018.A149, 81-02.04.2017.A406, 81-02.04.2013.A464 and 81-02.04.2010.A279 and performed in accordance with the ARRIVE guidelines. Mice were housed in cages under standard laboratory conditions (22–24°C temperature, 55%–60% relative humidity, and 12 h light/dark cycle), with standard food and water provided ad libitum. The hygiene monitoring took place quarterly according to the recommendations of the FELASA (Federation of European Laboratory Animal Science Associations) for the health monitoring of laboratory animal facilities. Animals were handled and housed according to specific pathogen free (SPF) conditions in the local breeding facility (ZETT, Zentrale Einrichtung für Tierforschung und Tierschutzaufgaben, Heinrich-Heine-University Düsseldorf).

Hepatocyte isolation and cultivation – After intraperitoneal injection of Ketavet (100 µg/g body weight) and Rompun (5 µg/g body weight) the abdominal cavity of the anesthetized mouse was opened. To remove the blood, the liver was perfused through the portal vein with HANKS buffer I (137 mM NaCl, 5 mM KCl, 15 mM Hepes, 0.34 mM $Na_2HPO_4x2H_2O$, 0.44 nM KH_2PO_4, 2 mM EGTA and 0.1 % [w/v] Glucose, pH 7.4, 37°C) using a peristaltic pump at a flow rate of 8 ml/min. To reduce the pressure the vena cava and the right heart ventricle were incised. After 2 min the perfusion with HANKS buffer II (37 mM NaCl, 5 mM KCl, 15 mM Hepes, 0.34 mM $Na_2HPO_4x2H_2O$, 0.44 nM KH_2PO_4, 0.1 % [w/v] Glucose, 5 mM $CaCl_2$ and 240 U/ml collagenase, pH 7.4, 37°C) was started until the liver swelled up. The liver was removed from the animal and transferred into a tube with William´s medium E (Biochrom) supplemented with 10 % fetal calf serum (FCS, Biochrom), 2 mM glutamine and 1 % penicillin/streptomycin (Gibco). In a sterile hood the capsule of the liver was carefully opened by a tweezers and the liver cells were flushing out by gentle shaking. The cell suspension was placed on a 70 µm cell strainer to remove tissue residues. The cells were washed once with William´s medium, then twice with autoMACS rinsing solution

supplemented with 0.5 % [w/v] BSA for separation by centrifugation (50 x g, 3 minutes, 20°C). After removing the last supernatant, antibodies against CD11b coupled to magnetic beats (Miltenyi Biotec, #130-049-601) were added to remove CD11b positive cells and incubated for 15 min at 4°C. The cell suspension was filled up to 10 ml by autoMACS rinsing solution and put on large cell separation columns (Miltenyi Biotec, #130-042-202) in a magnetic field (Fig. S1B,C). The cell number of vital hepatocytes in the flow through was determined by staining an aliquot with trypan blue.

The hepatocytes were cultivated in companion 6-well plates (BD Biosciences) with 0.8 Mio cells per well. To cultivate hepatocytes over several days under steroid-free conditions without the fibroblastoid dedifferentiation typical for the epithelial-mesenchymal transition of these cells, hepatocytes were embedded in a collagen matrix (Fig. S1F) [3]. Briefly, the plates were pre-coated with 350 µl collagen from rat tail (1 mg/ml collagen, Roche, #11179179001), Hepatocytes were plated in 2 ml Williams E Medium (supplemented with 10 % [v/v] FCS, 2 mM glutamine and 1 % [v/v] penicillin/streptomycin). After 3 h of incubation in a humidified atmosphere at 37 and 5 % CO_2, a second layer of 350 µl collagen was added to the washed cells. The hepatocytes were cultivated up to 7 days with Williams E Medium supplemented with 2 mM glutamine and 1 % [v/v] penicillin/streptomycin.

In comparison to the sandwich, culture (Fig. S1F), the cells were also cultivated as monolayers. Therefore, cells were plated on rat tail collagen I-coated tissue culture dishes with William’s medium E supplemented with 10 % [v/v] FCS, 100 nM dexamethasone, 2 mM L-glutamine and 1 % [v/v] penicillin/streptomycin. Cells were kept in a humidified atmosphere at 37 and 5 % CO_2 for 3 h to allow hepatocytes to attach. The cells were washed twice and cultured in medium without FCS (see above). Following incubation with this medium overnight, the experiments were started in starvation medium (William´s medium E supplemented with 2 mM L-glutamine and 1 % [v/v] penicillin/streptomycin).

Isolation of non-parenchymal liver cells and analysis by flow cytometry - For the isolation of NPLC, mouse livers were perfused with ice cold phosphate-buffered saline (PBS [w/o Ca/Mg]) and dissected mechanically. Hepatocytes and debris were sedimented twice at 40 × g, and NPLC were recovered by centrifugation over a 35% Optiprep (Sigma-Aldrich) gradient at 400 × g. The freshly isolated cells were stained with LIVE/DEAD dye Zombie (BioLegend) to exclude non-viable cells and subsequently fixed and permeabilized by 3% [v/v] Formaldehyd with Methanol. After removal of the Formaldehyd by adding FACS-buffer (PBS [w/o Ca/Mg] + 2% [v/v] FCS) and centrifugation (400 x g, 7 minutes, 4°C) the cells

were resuspended in FACS-buffer substituted with 25 µg/ml DNaseI (Roche) and TruStain FcX Plus (anti-mouse CD16/32, BioLegend) and incubated for 10 minutes in the dark on ice before the corresponding antibodies in sevenfold panels (antibodies are listed in Table S3A) were added and stained overnight at 4°C. After washing twice, F4/80 positive liver cells were identified by subtracting the corresponding fluorescence minus one (FMO) control by means of flow cytometry (FACS Canto II, BD Biosciences). Further different subpopulations were defined at the expression level of CD11b and CD14 and their respective fluorescence intensity of different markers was analyzed using the gating strategy delineated in Figure S2. Unstained, single color and FMO control tubes were used to generate compensation matrices that correct for spectral overlap. All data were analyzed by Flow Jo 10.6.7.

Isolation and differentiation of bone-marrow cells – The generation of BMDM was similar to the description of Rex et al. 2016 [4] with only a few changes: For isolation of bone-marrow cells both femurs and tibiae of scarified mice were removed and dissected free of adherent tissue. Bones were kept in PBS (w/o Ca/Mg, Biochrom) before incubation in 70 % [v/v] ethanol for 3–5 minutes. After short washing with PBS and transfer into rinse medium (DMEM 1 g/ml Glucose; Biochrom, Berlin, Germany, supplemented with 1% [v/v] Penicillin/Streptomycin; PAN Biotech, Aidenbach, Germany) both ends of the bones were cut and the bone marrow was flushed out by irrigation with rinse medium by a syringe (needle: 23G x 1“). A single cell suspension was obtained by pipetting cells vigorously followed by centrifugation (300 x g, 4°C, 10 minutes). Cells were resuspended in culture medium (DMEM 1 g/ml Glucose, 1% [v/v] Penicillin/Streptomycin, 10% [v/v] FCS). Cells were cultured in a 175 cm^2 cell culture flask overnight. Non-adherent cells were collected in a 50 ml Falcon and harvested by centrifugation. Cells were resuspended in culture medium, supplemented with 10 ng/ml murine M-CSF (mM-CSF) (Peprotech) and cultivated in five cell culture dishes with 15 cm diameter and 20 ml medium (including mM-CSF). 20 ml of medium supplemented with mM-CSF was added on day 3 and 10 ml on day 6 of cultivation (without aspiration of the old media). After 7 days cells were washed twice with PBS and treated with 3 ml trypsin/EDTA (PAN Biotech) for approximately 10 minutes. Cells were centrifuged and resuspended in medium with mM-CSF. After evaluation of the total cell number, 0.1 Mio BMDM per transwell (BD Biosciences) were seeded in medium with mM-CSF.

Co-culture – One day after preparation of primary hepatocytes and splitting BMDM the

supernatant of hepatocytes and from control wells (no hepatocytes, only collagen) was removed. Per well 2 ml starvation medium was added. The supernatant of the transwells was also removed and the transwells were transferred to the 6-well plates containing hepatocytes or to control wells. Per transwell 2 ml starvation medium containing 10 ng/ml mM-CSF was added.

For FACS-analysis BMDM were mono- or co-cultivated for up to four days. For stimulation with TGF-β1 (5 ng/ml) it was given once, and cells were cultured for three days. Anti-TGF-β1, 2, 3 (0.25 µg/ml) was given daily for three days or cells were stimulated with ECM1 (25 ng/ml) for 24 hours. For cell harvest the transwells were put into a new 6-well plate and washed twice with PBS. After incubation with 0.5 ml Trypsin for 5 minutes, cells were rinsed off by pipetting and collected by centrifugation (450 x g, 5 minutes, 4°C). BMDM were stained with antibodies (Tab. S3B) in the dark on ice for 1 hour and analyzed by FACS Canto II (BD Biosciences).

For basal mRNA expression analysis BMDM were mono- or co-cultivated for one to three days. For LPS-stimulation, the cells were cultivated for two days and then stimulated with 10 ng/ml LPS for indicated time points. For RNA isolation, the transwells were put into a new 6-well plate, washed twice with PBS and lysed by RLT-buffer.

RNA isolation, cDNA synthesis and real-time PCR – RNA was isolated with the RNeasy Mini Kit (Qiagen) according manufacturer's instructions. The cDNA synthesis was performed with the QuantiTect Reverse Transcription Kit (Qiagen). Quantitative real-time (rt)PCR was performed with GoTaq qPCR Master Mix (Promega) as described in the manufacturer's instructions. The primers used are listed in Table S5. The cDNA amplification was carried out over 40 cycles by ViiA7 Real-Time PCR System (ThermoFisher). Data were produced in duplicates for each gene. Semi quantitative PCR results were calculated using the delta-delta CT method and threshold values were normalized to Succinate Dehydrogenase Complex Flavoprotein Subunit A (Sdha) or hypoxanthine phosphoribosyltransferase-1 (Hprt).

Cytokine detection in the supernatant – IFN-beta ELISA (#42400, pbl Assay Science), IL-10 ELISA (#88-7105-88, eBioscience) and TGFb1 ELISA (#436707, BioLegend) as well as Luminex assays (#TGFBMAG-64K-03 and #MYCTOMAG-70K, Merck Millipore) were used to analyze cytokine levels in (concentrated) supernatants according to manufacturer's instructions.

Concentrating of supernatant – For detection of IL-10 and TGF-β1 active the supernatant of unstimulated mono- and co-cultivated BMDM was collected and centrifuged for 20 minutes by maximum speed and 4°C to remove cell debris. The supernatant was concentrated by Amicon Ultra centrifugal filters (pore size 3 K) (Merck Millipore) about 16-fold. The weight of the concentrated supernatant was detected to calculate the initial concentration of TGF-β1 active and IL-10.

Mass spectrometric analysis of hepatocytes and BMDM (proteome) as well as of the supernatant (secretome) – Hepatocytes and BMDM from mono- and co-cultures were washed with PBS, harvested using a rubber-policeman, proteins extracted from cell pellets with lysis puffer (30 mM Tris-HCl; 2 M thiourea; 7 M urea; 4 % [w/v] CHAPS, pH 8.0) and prepared for mass spectrometric analysis essentially as described earlier [6]. Briefly, 5 µg of proteins per sample were shortly stacked in a polyacrylamide gel (about 5 mm running distance), subjected to silver-staining, reduced with dithiothreitol, alkylated with iodoacetamide and finally digested with trypsin.

Resulting peptides were extracted from the gel, vacuum-dried and 500 ng per sample reconstituted in 0.1 % trifluoroacetic acid for subsequent liquid chromatography coupled mass spectrometric analysis.

First, an Ultimate 3000 Rapid Separation liquid chromatography system (Thermo Scientific, Dreieich, Germany) was used for peptide separation. After pre-concentration of peptides on a trap column (Acclaim PepMap100, 3 µm C18 particle size, pore size 100 Å, inner diameter 75 µm, 2 cm length, Thermo Scientific, Dreieich, Germany) for ten minutes at a flow rate of 6 ml/min using 0.1 % [v/v] TFA as mobile phase, peptides were separated on an analytical column (Acclaim PepMapRSLC, 2 µm C18 particle size, 100 Å pore size, 75 µm inner diameter, 25 cm length, Thermo Scientific, Dreieich, Germany) at 60°C using a 2 h gradient from 4 to 40 % solvent (0.1 % [v/v] formic acid, 84 % [v/v] acetonitrile in water) at a flow rate of 0.3 µl/min.

Separated peptides were directly injected in an online coupled Orbitrap Elite high resolution hybrid mass spectrometer (Thermo Scientific, Bremen, Germany) via a nano electrospray ionization source using distal coated silica emitters (FS360–20–10-D; New Objective, Woburn, MA, USA). The mass spectrometer was operated in positive mode; capillary temperature was set to 275°C and source voltage to 1.5 kV. Full scans were recorded in the orbitrap analyzer in profile mode with a resolution of 60,000 over a scan range from 350 to 1,700 m/z with a maximum ion time of 200 ms and the target value for the automatic gain

control set to 3,000,000. Up to 20 two- and threefold charged precursor ions were isolated in the linear ion trap within a 2 m/z isolation window, fragmented via collision induced dissociation and fragments analyzed with a maximal ion time of 50 ms and the target value for the automatic gain control set to 30,000. The resolution was set to 5,400, the available scan range was 200 to 2,000 m/z and spectra were recorded in centroid mode. Already fragmented precursors were excluded from analysis for the next 45 s.

Recorded spectra were further processed for protein identification and quantification with Maxquant 1.5.3.30 (Max Planck Institute of Biochemistry, Planegg, Germany) using standard parameters if not otherwise stated. Database searches were carried out using 51414 sequence entries downloaded on the 20th June 2016 from the UniProtKB mus musculus proteome section (UP000000589) considering carbamidomethylation at cysteines as fixed and methionine oxidation and acetylation at protein N-termini as variable modifications. Label-free quantification was enabled as well as the “match between runs” function. Peptides and proteins were accepted at a false discovery rate of 1% and only proteins considered for further analysis showing at least two different identified peptides and at least four valid values in at least one analysis-group.

Three different sets of searches were carried out: First hepatocytes (mono and co-culture), second macrophages (mono and co-culture) and third hepatocytes and macrophages (each mono and co-culture).

For the volcano plots quantitative data was further analyzed using MSPypeline [7] and p-values, from a pairwise comparison of two groups, were determined using the R limma package. Calculations were corrected for the intensity-variance relationship.

The mass spectrometry proteomics data have been deposited to the ProteomeXchange Consortium via the PRIDE [8] partner repository with the dataset identifier PXD012136.

Microarray Assays - The totalRNA samples used for transcriptome analyses were quantified and quality measured by capillary electrophoresis Bioanalyzer assay (Eukaryote Total RNA Pico, Agilent). All samples in this study showed high quality RNA Integrity Numbers (RIN; mean = 9.7). Synthesis of cDNA and subsequent biotin labeling of cRNA was performed according to the manufacturer’s protocol (GeneChip® Pico Reagent Kit 703308 Rev. 4; ThermoFisher scientific). Briefly, 1 ng of total RNA were converted to cDNA, amplified to complementary RNA (cRNA) followed by in vitro transcription and biotin labeling of cDNA. After fragmentation labeled cDNA was hybridized to Applied Biosystems™ Clariom™ S Mouse Gene Expression Microarrays for 16 hr at 45°C, stained by streptavidin/phycoerythrin

conjugate, and scanned as described in the manufacturer's protocol.
Data analyses on Affymetrix CEL files were conducted with GeneSpring GX software (Vers. 12.5; Agilent Technologies). Probes within each probe set were summarized by GeneSprings' ExonRMA16 algorithm after quantile normalization of probe-level signal intensities across all samples to reduce interarray variability [9]. Input data pre-processing was concluded by baseline transformation to the median of all samples. After grouping of samples (five biological replicates each) according to their respective experimental condition, a given probe set had to be expressed above background (i.e., fluorescence signal of that probe set was detected within the 20th and 100th percentiles of the raw signal distribution of a given array) in 75 % of the replicates in at least one of the four conditions to be further analyzed in pairwise comparisons. Differential gene expression was statistically determined by Moderated t test. The Resulting *P* values were corrected for multiple testing by FDR-correction. A *P* value of ≤0.05 was considered significant. Hierarchical cluster analysis was performed with Euclidian similarity measures and Ward's linkage. The data was further evaluated with the Ingenuity-Pathway analysis software (Qiagen Inc. 2016). Microarray RNA data have been deposited in GEO with the dataset identifier GSE201467.

Fluorescence Activated Cell Sorting for single-cell library generation - For single cell analysis the livers were perfused with DMEM until they were bloodless. NPLC were isolated with the Liver Dissociation Kit from Miltenyi Biotec according to the manufacture instructions. The cells were blocked by Fc-Block (1:200, True stain FcX Plus Biolegend) for 10 minutes on ice, than incubated on ice with Zombie (1:1000, Biolegend), CD45 (1:100, Biolegend) and F4/80 (1:100, Biolegend) in PBS with 2 mM EDTA for 30 minutes. The cells were washed and vital, CD45 positive, F4/80 positive cells were sorted using a BD FACS Aria III SORP Cell sorter (BD Biosciences) and a 100 µm nozzle.

Single-cell experiments, 10x sample processing, library preparation and sequencing - For single-cell experiments the NPLC of 2 TGF-βRII^{flox} and 2 TGF-βRII$^{\Delta MC}$ mice were isolated as described above. Single cell processing was carried out on the 10X Chromium Controller system utilizing the Chromium Single-Cell 3'NextGEM Reagent-Kit-v3.1 (10X Genomics, Pleasanton, CA) aiming for an average recovery of 10,000 cells per sample. Sequencing was carried out on aNextSeq-550 system (Illumina Inc. San Diego, CA) with a mean sequencing depth of ~50,000 reads/cell.

Processing of 10X Genomics single cell data - Raw sequencing data was processed using the 10X Genomics Cell Ranger software (v6.0.0). Raw BCL-files were demultipexed and processed to Fastq-files using the CellRanger mkfastq pipeline. Alignment of reads to the mm10 genome and UMI counting was performed via the CellRanger count pipieline to generate a gene-barcode matrix. All samples were aggregated and normalized for sequencing depth using the cellranger aggr pipeline. Further analyses were carried out with the Seurat v4.0.5 R package [10-12]. Initial quality control was performed for each sample and consisted of removal of cells with less than 200 detected genes and less than 500 unique molecular identifies. Additionally, cells belonging to the top 1 % based on the number of genes were discarded and genes expressed in less than 3 cells were removed. Furthermore, cells with a mapping rate of > 10 % to the mitochondrial genome were removed, as they represent dead or damaged cells. Cell doublets were removed from the dataset using DoubletFinder v2.0 with default parameters [13]. Normalization has been carried out using the method LogNormalize in Seurat. Samples were then integrated by selecting the top 1,500 most recurrent variable genes across all samples. PCA correction was performed with harmony [14] using sample and mouse type labels as covariates. Dimensional reduction of the data set was achieved by Principal Component analysis (PCA) based on the 3,000 most variable features and subsequent uniform manifold approximation and projection (UMAP) embedding using the first 30 principal components (PCs). With the same PCs a shared nearest neighbour graph was build using FindNeighbors and cells were clustered using the Louvain algorithm implemented in Seurat. Markers defining each cluster as well as differential gene expression between different clusters were calculated using a Wilcoxon Rank Sum test implemented in Seurat. Finally, cells were assigned to major cell lineages by manually inspecting cluster marker genes and confirmed by performing an enrichment analysis using hypergeometric tests with cell type markers from Aizarini et al. [15]. RNA-seq data have been deposited in the ArrayExpress database at EMBL-EBI (www.ebi.ac.uk/arrayexpress) under accession number E-MTAB-11727.

Partial hepatectomy - $^{2}/_{3}$ PHx was performed as described previously [5]. For sham operation, a midline abdominal skin and muscle incision was performed. After gently pulling the left and middle flaps with a cotton tip moistened with saline, the peritoneum and the skin were closed again by suturing.

Serum biochemistry - Aspartate aminotransferase (AST), alanine aminotransferase (ALT),

Lactate dehydrogenase (LDH), Albumin, total Bilirubin, total Protein and Alkaline phosphatase (ALP) were measured in the serum using the automated biochemical analyzer Spotchem EZ SP-4430 (Arkray) and the Spotchem EZ Reagent Strips Liver-1 and ALP.

Preparation of cryosections, immunofluorescent staining and microscopy - 10 µm thick cryosections from the tissue of the left lobe of the murine liver were prepared using the cryotome CM3050S (Leica) and attached to object slides. Antibodies used for staining are listed in Table S4.

A) *TGFb-LAP Staining* – Sections at room temperature were fixed by 4% PFA for 15 minutes, washed three times with PBS and permeabilized and blocked with 5% FBS and 0.3% Triton X-100 in PBS for one hour. The slides were incubated overnight at 4°C with Anti-TGFb-LAP (1:100 in 1% BSA and 0.3 Triton X-100 in PBS) from Cosmo Bio. After washing three times the sections were incubated with the secondary antibody (AlexaFluor 488 AffiniPure F8 (ab')2 Fragment Donkey Anti-Mouse IgG (H+L), 1:200, Jackson ImmunoResearch) and DRAQ5 (1:1000. Cell signaling) in 1% BSA and 0.3% Trition X-100 in PBS for one hour at room temperature. After washing again three times the slices were mount with Dakocytomation fluorescent mounting medium (DAKO). Stained samples were analyzed by microscopy using the TCS SP8 upright, Confocal Microscope from Leica.

B) *Proliferating cell staining* - Tissue slices were fixed with methanol at -20 °C for 5 minutes. Thereafter, samples were washed four times at room temperature with PBS w/o $Ca2^+$ or $Mg2^+$ and then blocked with 5% BSA for one hour. The tissue slices were incubated with primary antibodies targeting Ki67 (Abcam, rabbit-anti-mouse), F4/80 (Bio-Rad, rat-anti-mouse) and CD26 (R&D Systems, goat-anti-mouse) diluted 1:200 in blocking buffer for 90 minutes. After additional washing four times with PBS, slices were incubated with fluorochrome-conjugated secondary antibodies diluted 1:200 in blocking buffer (FITC-labeled donkey-anti-rabbit, Cy5-labeled donkey-anti-rat, Cy3-labeled donkey-anti-goat, Dianova) and Hoechst 33258 staining solution diluted 1:20,000 (Sigma-Aldrich). Following a four times washing step with PBS, tissue slices were covered with mounting medium (Dako/Agilent) and cover slips. Stained samples were analyzed by microscopy using the cell observer system from Zeiss. 15 color images per animal were recorded with a 25-fold magnification.

Automated counting of immunofluorescent stained cells - The abundance of Ki67 and

Hoechst 33258 positive cells within the tissue slices was assessed by automated counting of immunofluorescent stained cells using the ImageJ 1.52n Fiji software from the National Institutes of Health (USA). Thereby, for cell counting each image with 684 x 516 pixels in size was converted into 16-bit greyscale. Subsequently, a binary version of the image was created with only black and white color by adjusting manually a threshold between the pixels of interest from positive-stained cells and the background. Hereby, the structures to count were highlighted in white on a black background. Thereafter, white objects were counted using the “analyze particles” option of the software excluding objects smaller than 50 square microns. For each signal (Ki67 or Hoechst 33258) the mean value of all 15 images per animal represented the amount of positive cells per image in one animal.

Statistical evaluation - Statistics were calculated using the GraphPad Prism software (Prism 5). Since normal distribution cannot be assumed for the data and the random number the Mann-Whitney-U Test was used as nonparametric T-test. mRNA Data are expressed as fractions of the normalised value of a control, which was set to 1. Data are presented as mean ± SEM. The number of independent experiments underlying the data depicted is provided within the respective figure. P values smaller than 0.05 were considered significant. Data were marked with * for $p \leq 0.05$, ** for $p \leq 0.01$ or *** for $p \leq 0.001$ for differences between two data series and with # for $p \leq 0.05$ differences to the control value of each data series. For sake of clarity, only significances that are relevant for the statement made by the figure are indicated.

The statistical analysis of the mass spectrometric analysis, the microarray assay and the single cell analysis is described in detail in the section of each method.

Supplemental Figures

Fig. S1

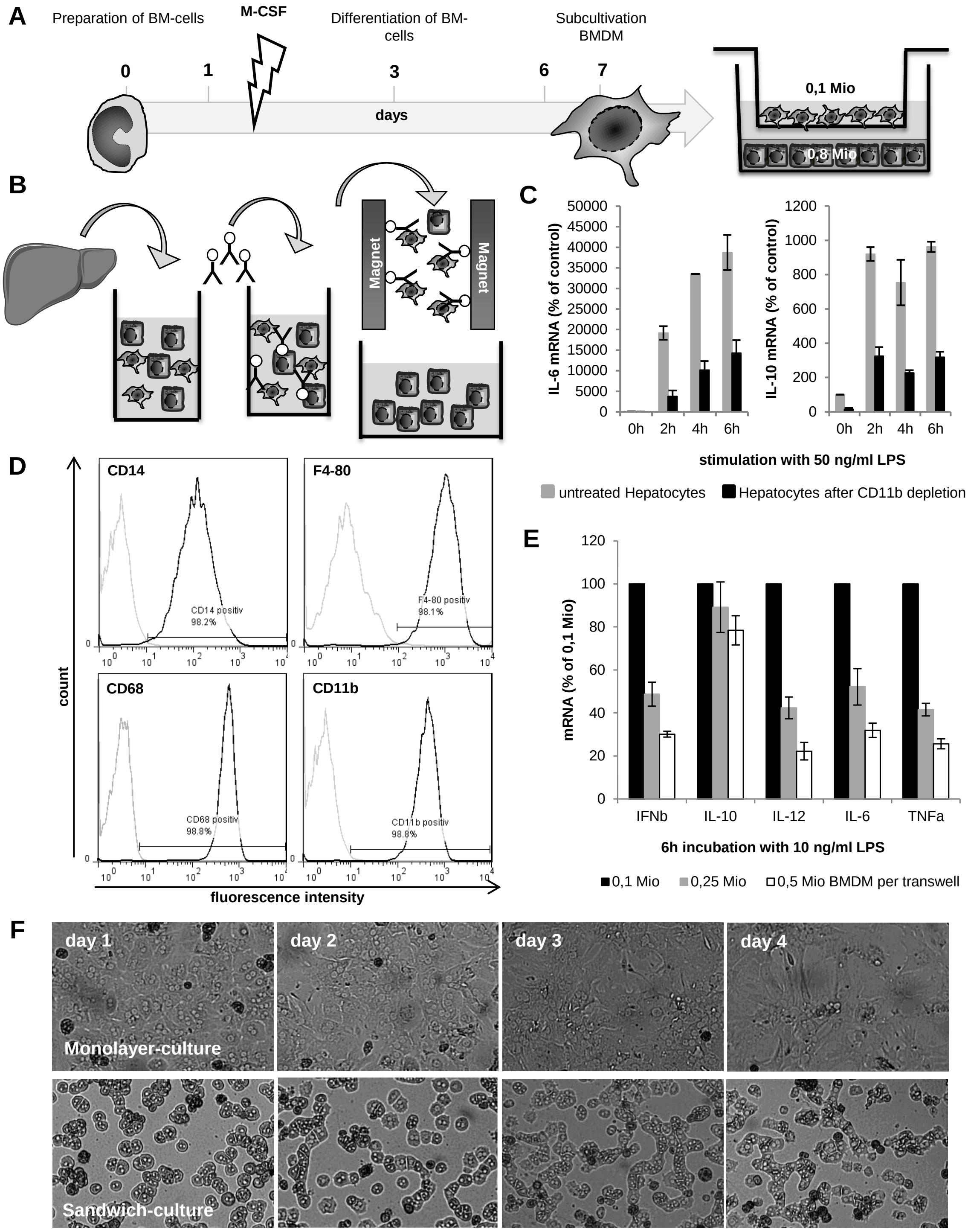

Fig. S1. Establishment of a co-culture system for BMDM and primary hepatocytes. (A, B) Schematic view of cell preparation and cultivation of BMDM and hepatocytes. (C) Comparison of IL-6 and IL-10 mRNA expression in primary hepatocytes untreated (grey) and

after depletion of CD11b positive cells (black) after stimulation with 10 ng/ml LPS (n=3). (D) Fluorescence intensity of CD14, F4/80, CD68 and CD11b (black) in BMDM after 7 days of differentiation in comparison to the respective isotype-controls (grey). (E) Influence of the cell density of BMDM on the mRNA expression of INFb, IL-10, IL-12, IL-6 and TNFa 6 hours after stimulation with 10 ng/ml LPS (n=3). (F) Primary hepatocytes in a single collagen layer culture (above) or in sandwich-culture (below) for 1 to 4 days. The data are presented as means ± SEM.

Fig. S2

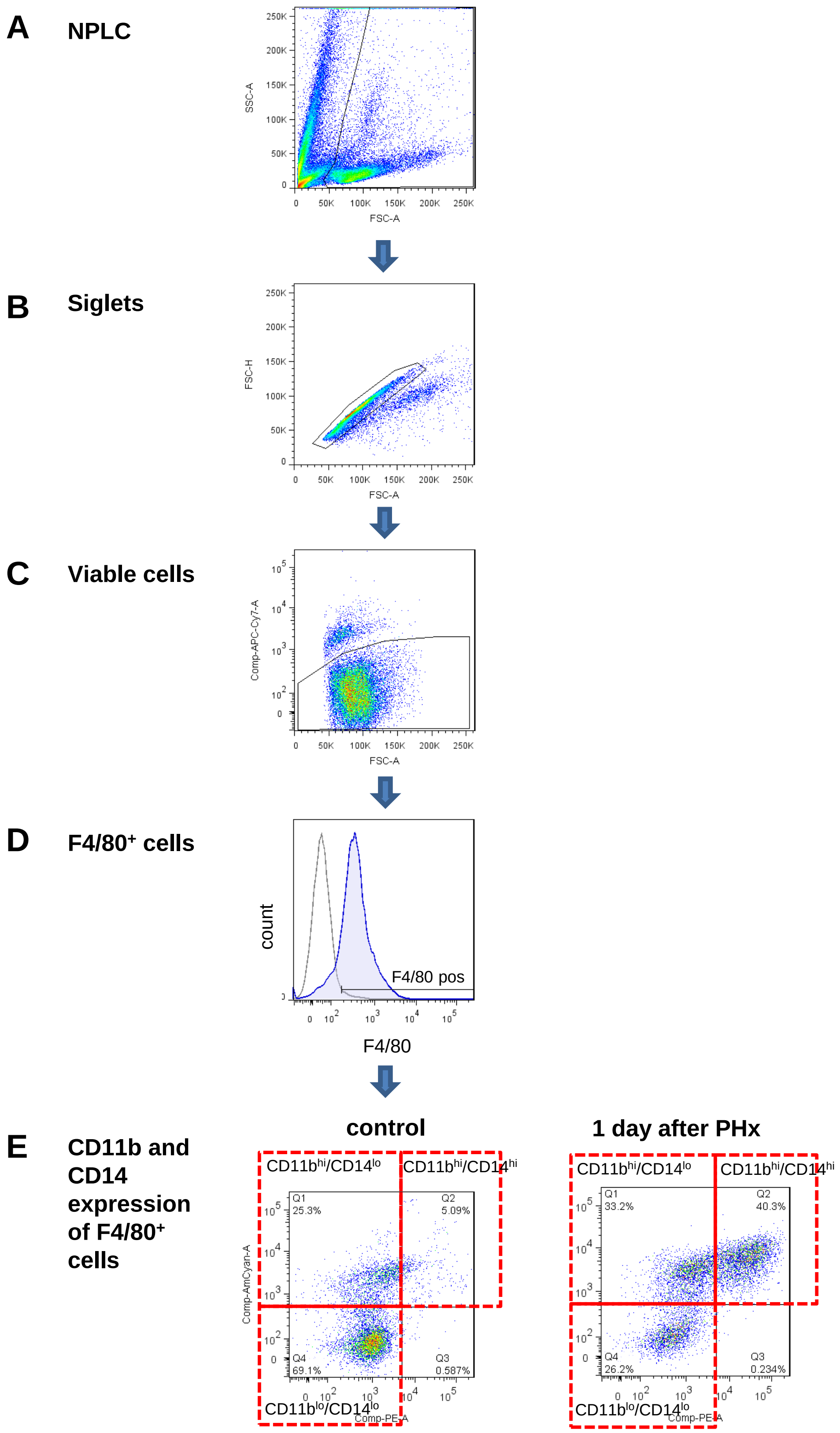

Fig. S2. Gating strategy for viable F4/80 positive liver cells and their CD11b and CD14 expression. (A) Removement of debris and cell fragments by gating the NPLC. (B) Removement of cell doublets and (C) identification of viable cells by staining dead cells with

Zombie. (D) Identification of F4/80 positive cells with an Antibody against F4/80 (blue) vs. FMO control (grey). (E) Differentiation of CD11b and CD14 high (hi) and low (lo) expressing F4/80 positive cells in control animals and one day after PHx.

Fig. S3

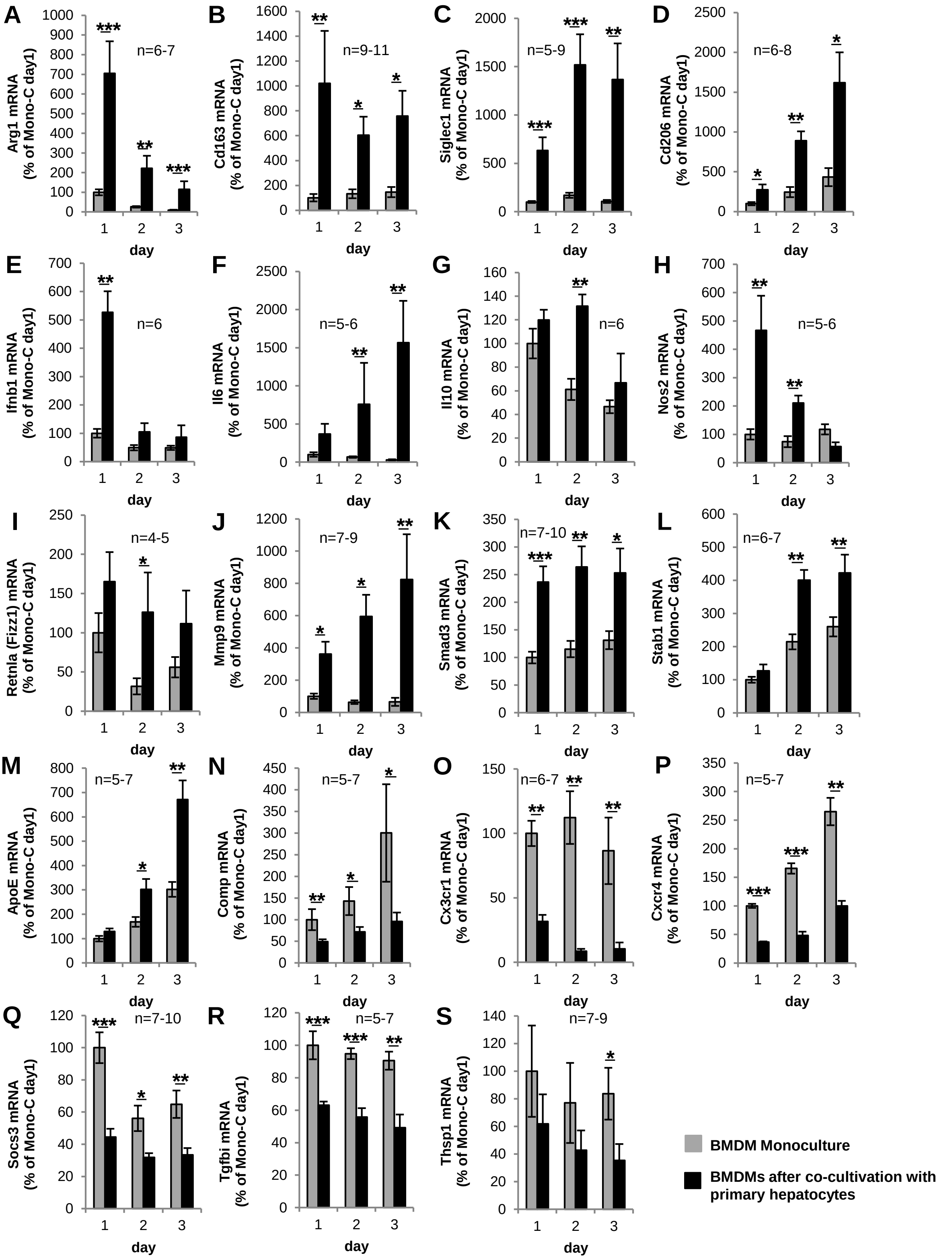

Fig. S3. Co-cultivation with hepatocytes results in an upregulation of markers that suggest alternative activation of BMDM. (A-S) Differences in analyzed parameters on

mRNA expression levels between mono- (grey) and with hepatocytes co-cultivated (black) BMDM after one to three days of cultivation. The data are presented as means ± SEM (number of replications are shown in the picture). Significant differences between mono- and co-culture are indicated by * for $p \leq 0.05$, ** for $p \leq 0.01$ and *** for $p \leq 0.001$ (Mann-Whitney U test).

Fig. S4

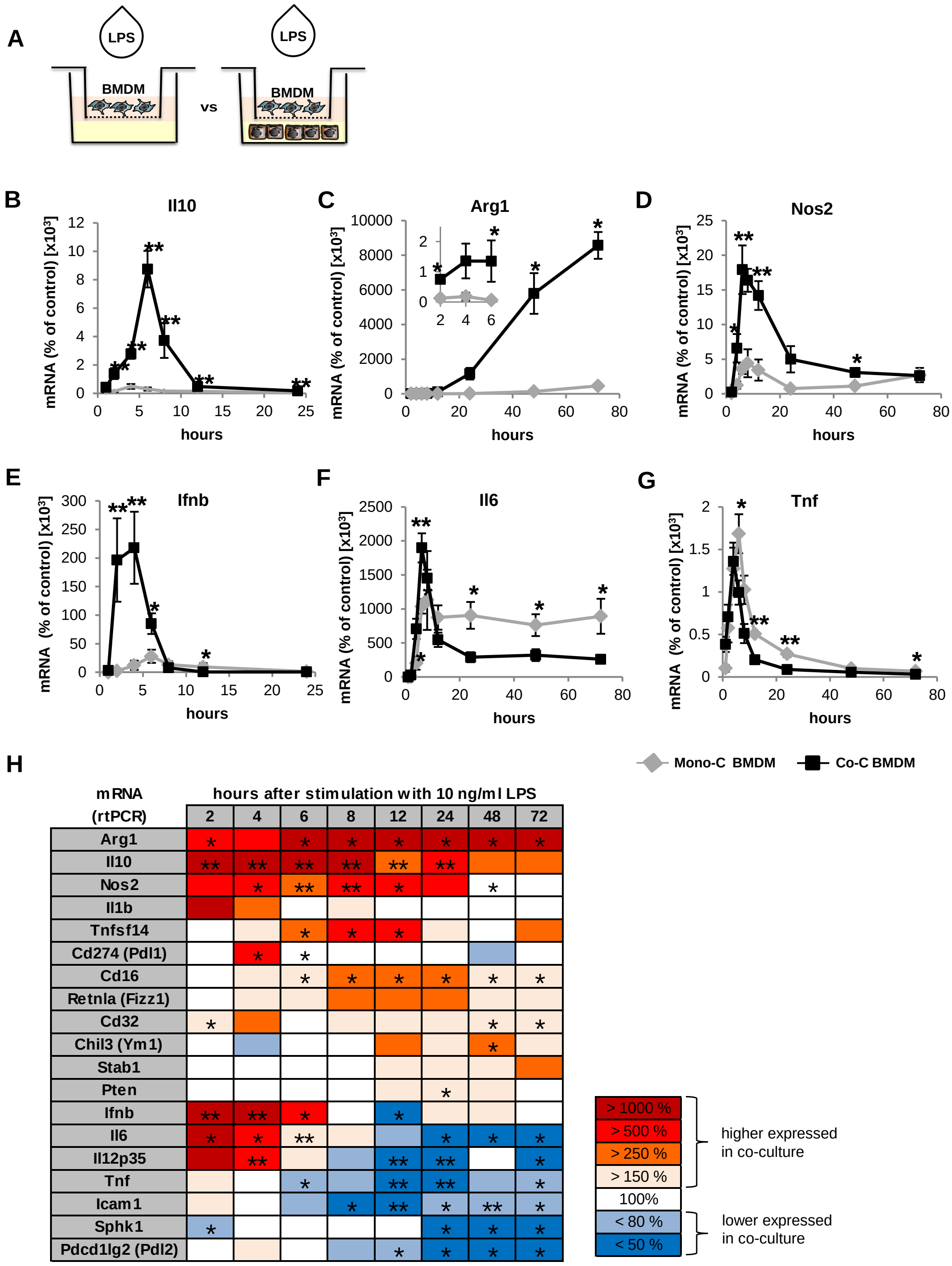

Fig. S4. Hepatocyte-cultured BMDM show a predominant anti-inflammatory phenotype upon stimulation with LPS. (A) Schematic presentation of the co-culture system. (B-H) Differences in the mRNA expression of analysed parameters between mono- and co-cultivated BMDM after 2 days of cultivation and subsequent stimulation with 10 ng/ml LPS for 2 to 72 hours (n=3-6). The data are presented as means ± SEM. Significant differences between mono- and co-culture are indicated by * for $p \leq 0.05$ and ** for $p \leq 0.0$ (Mann-Whitney U test).

Fig. S5

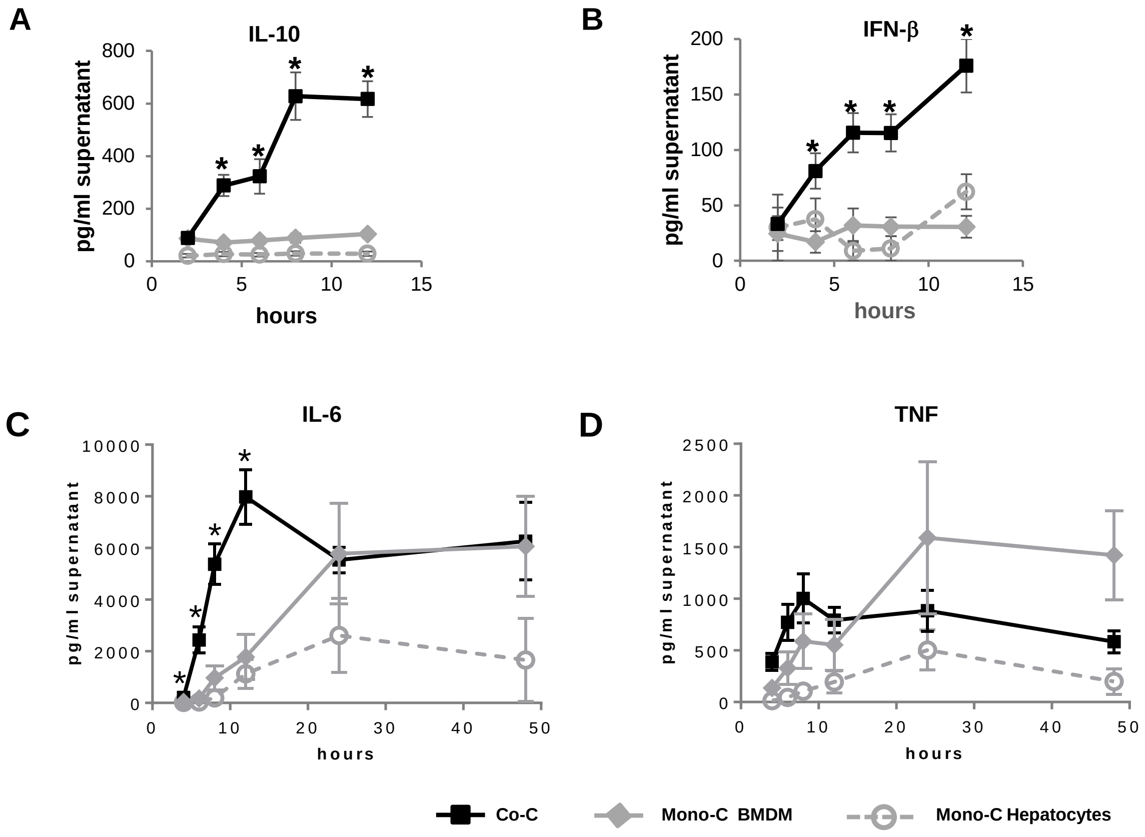

Fig. S5. Hepatocyte-cultured BMDM secrete predominant anti-inflammatory cytokines upon stimulation with LPS. (A-D) Protein concentration of IL-10, IFN-b, IL-6 and TNFα in the supernatant of mono- (gray rectangles) and with hepatocytes co-cultivated (black squares) BMDM or mono-cultivated hepatocytes (gray circles) after stimulation with 10 ng/ml LPS for up to 72 hours (n=3-7). The data are presented as means ± SEM. Significant differences between monoculture BMDM and co-culture are indicated by * for $p \leq 0.05$ (Mann-Whitney U test).

Fig. S6

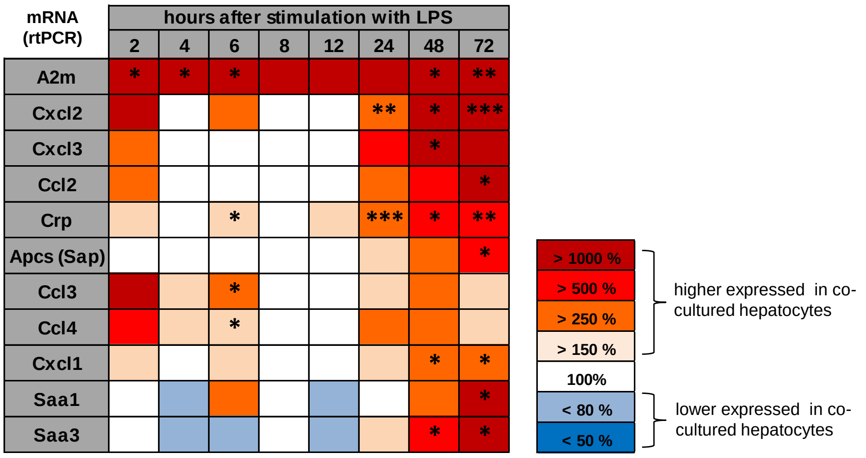

Fig. S6. Heatmap of mRNA-Expression of different chemokines and Acute-Phase Proteins in mono- and co-cultivated hepatocytes after stimulation with 10 ng/ml LPS. The data are presented as means ± SEM (n=5-7). Significant differences between mono- and co-culture are indicated by * for $p \leq 0.05$, ** for $p \leq 0.01$ and *** for $p \leq 0.001$ (Mann-Whitney U test).

Fig. S7

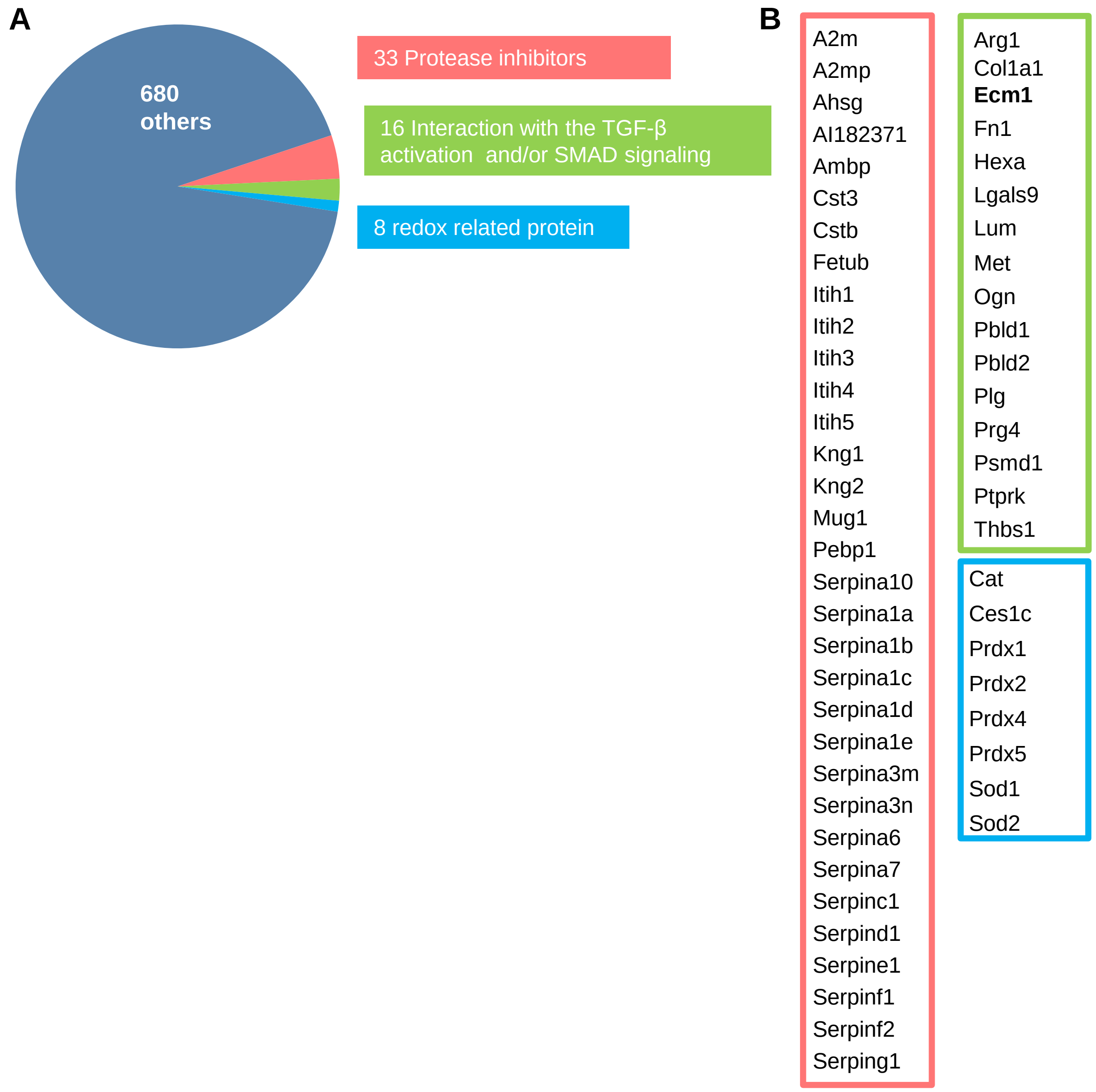

Fig. S7. Analyze of the secretome of mono-cultivated hepatocytes. (A) Pie chart of proteins which could be involved in TGF-β activation and / or signaling. (B) List of proteins released from primary hepatocytes which act as protease inhibitors (red), redox related protein (blue) or which interact with the TGF-β and / or SMAD signaling (green).

Fig. S8

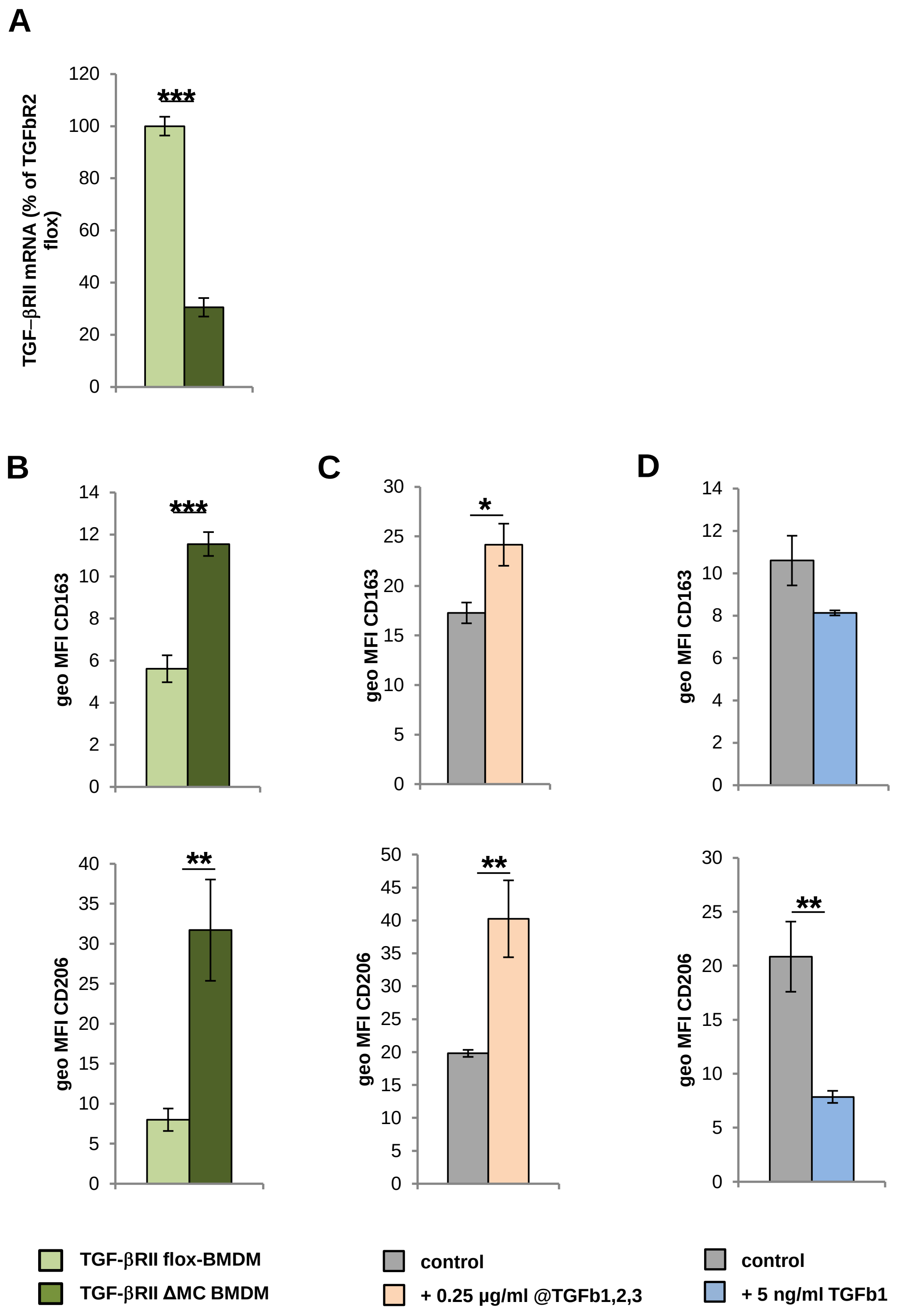

Fig. S8. TGF-β reduces the expression of CD163 and CD206 in BMDM. (A) Expression of TGF-βRII in BMDM isolated from TGF-βRII flox or TGF-βRII ΔMC mice (n=4) (B) Protein expression of CD163 and CD206 in TGF-βRII-depleted (dark green) and TGF-βRII^{flox} (light green) BMDM (n=7). (B) Expression of CD163 and CD206 after incubation of

wt mono-cultivated BMDM with an antibody against TGF-β1,2,3 (orange) (n=5) or (C) with TGF-β1 (blue) (n=6). The protein expression was detected by flow cytometry. The data are presented as means ± SEM. Significant differences between mono- and co-culture are indicated by * for $p \leq 0.05$, ** for $p \leq 0.01$ and *** for $p \leq 0.001$ (Mann-Whitney U test).

Fig. S9

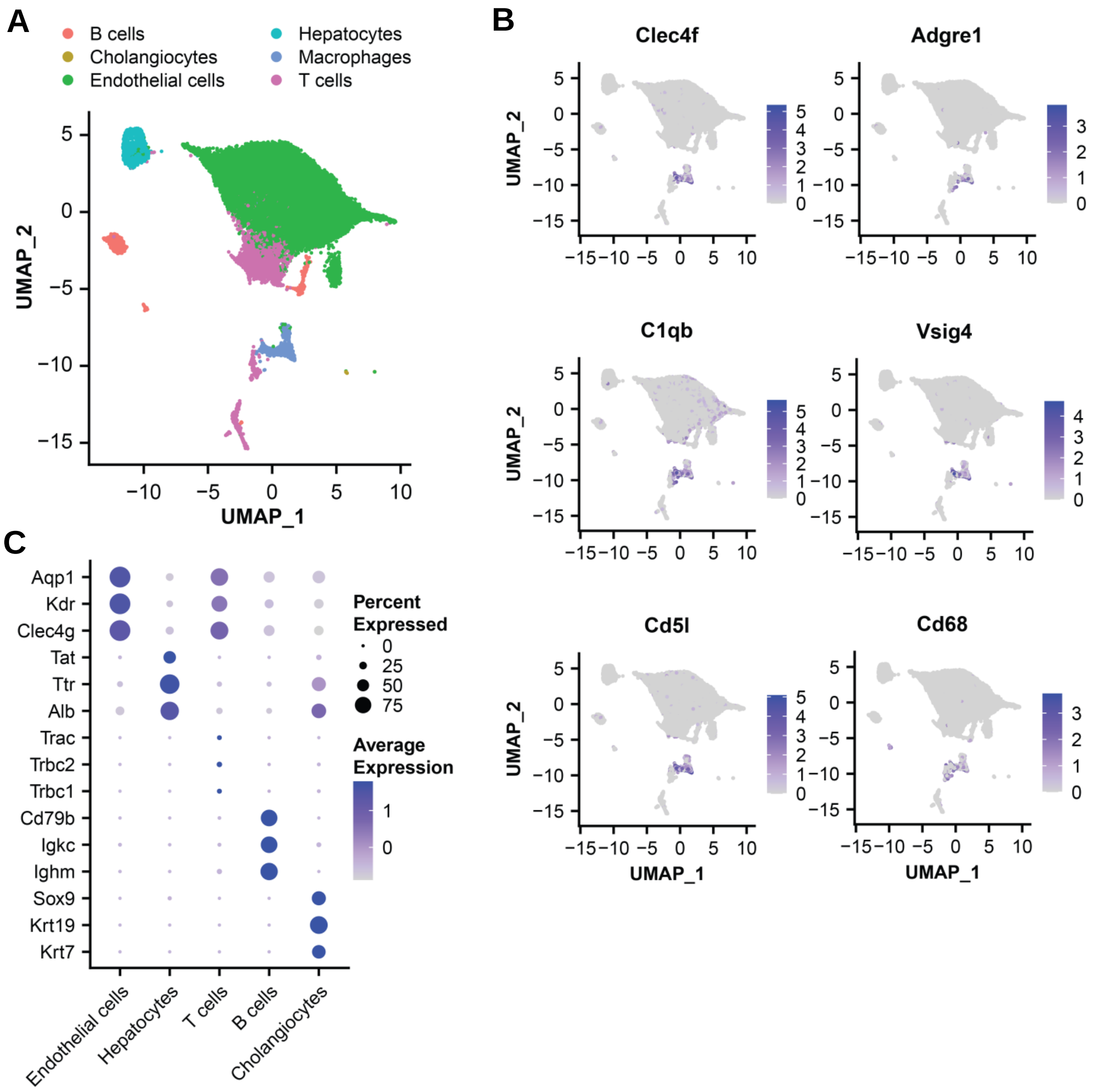

Fig. S9. Single cell data related to Figure 7. NPLC were enriched from whole liver lysates by FACS. (A) UMAP visualization of cells from TGF-βRIIflox and TGF-βRII$^{\Delta MC}$ mice (n = 2 per genotype) separated by their gene expression. (B) UMAP plots showing the expression of the macrophage markers Clec4f, Adgre1 (F4/80), C1qb, Vsig4, Cd5l and CD68. (C) Identification of the other cell populations that are leadingly characterized by the indicated gene expression. The color scale indicates the log-normalized gene expression level.

Fig. S10

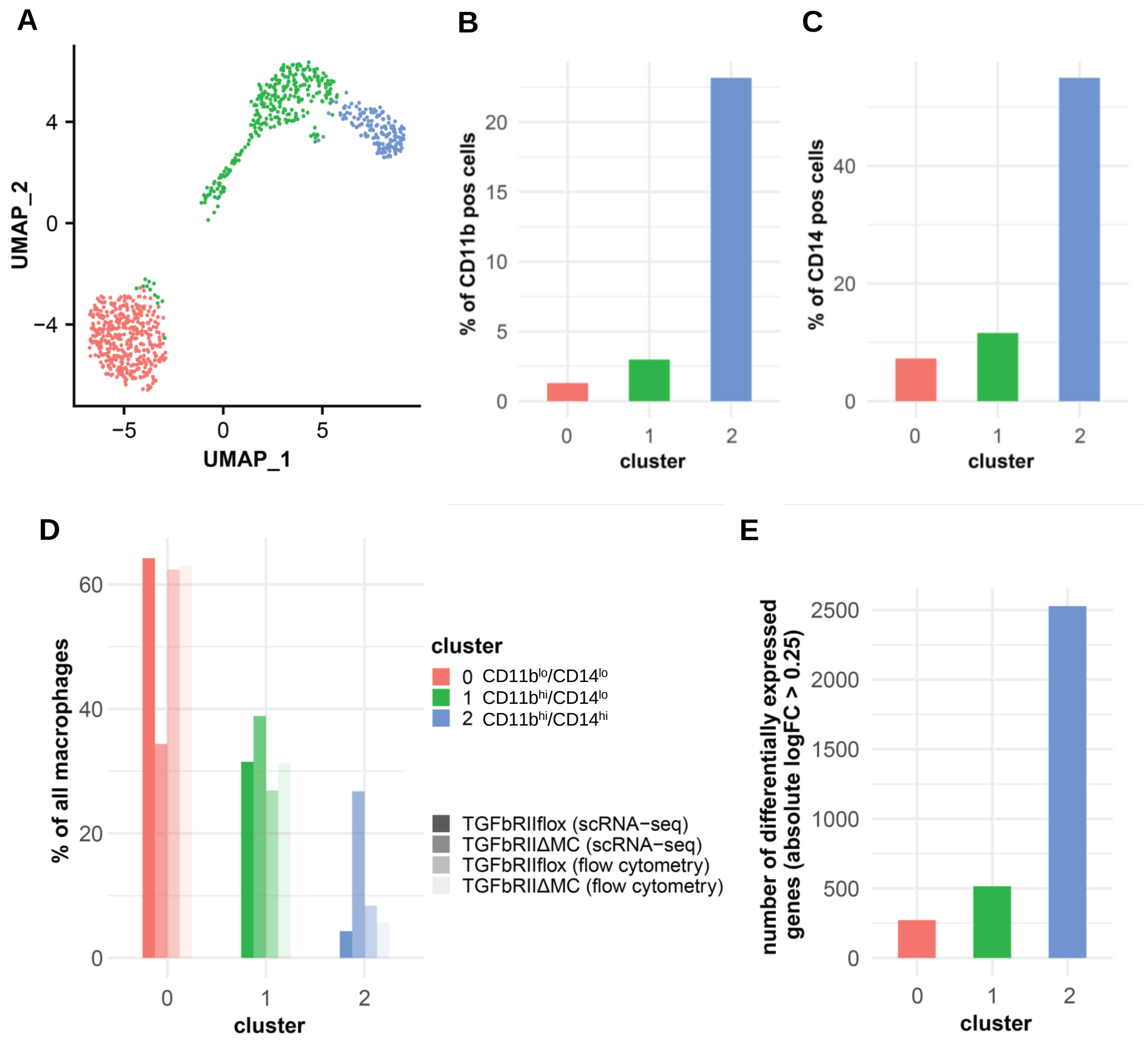

Fig. S10. Characterization of the macrophage subpopulations from the data of the single-cell RNA analysis. (A) UMAP visualization of macrophage subclusters according to their gene expression. (B) Percentage of CD11b and (C) CD14 positive macrophages in the different clusters. (D) Percentage of the individual clusters in the total macrophage population, separated according to their genotype (scRNA-seq) in comparison to the macrophage subpopulations defined by flow cytometry (Fig. 1). (E) Number of genes differentially expressed in TGF-βRII^{flox} and TGF-βRII$^{\Delta MC}$ macrophages in the different clusters (absolut_log2FC greater than 0.25).

Fig. S11

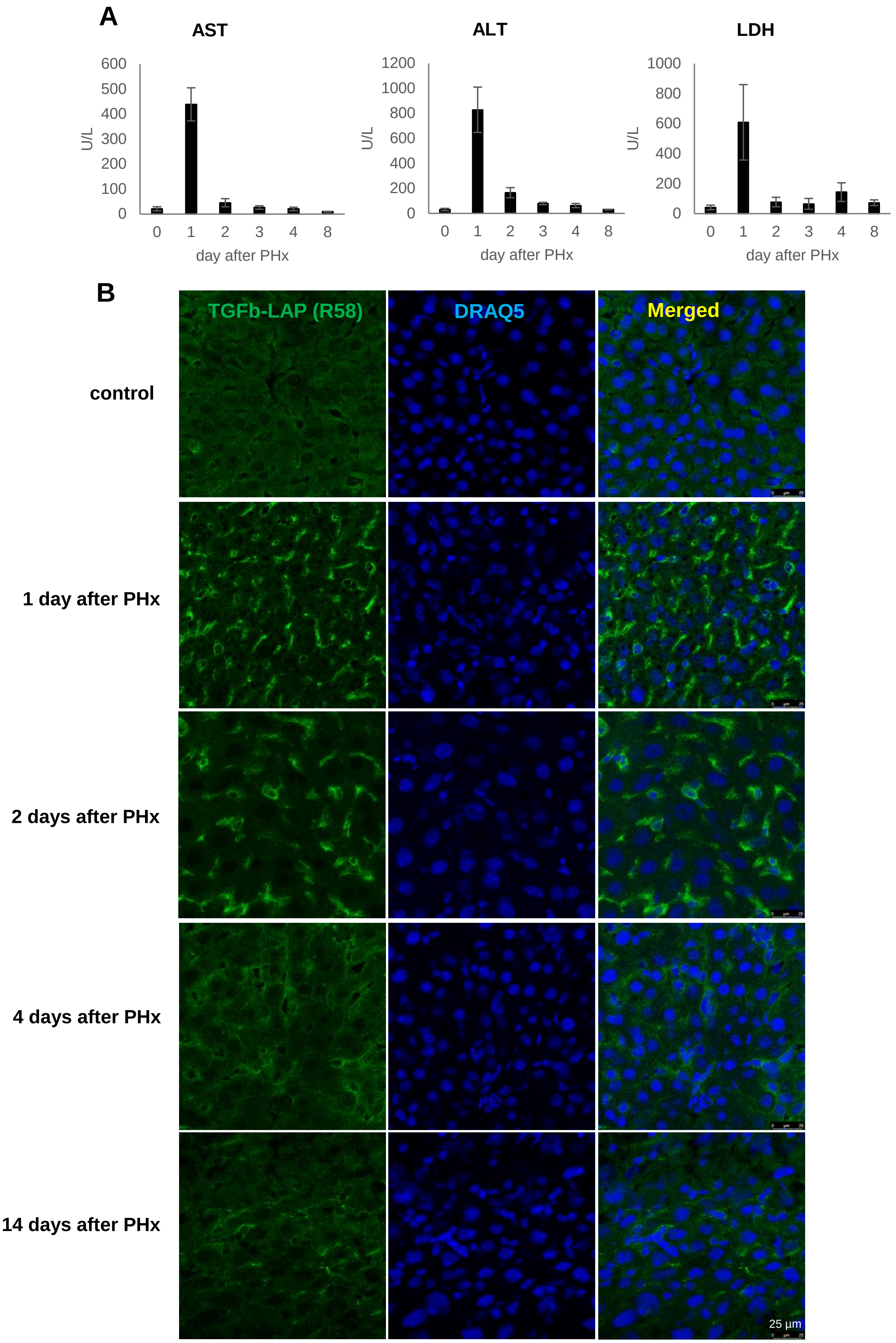

A
AST
ALT
LDH
U/L
600
500
400
300
200
100
0
1200
1000
800
600
400
200
0
1000
800
600
400
200
0
0 1 2 3 4 8
day after PHx
B
TGFb-LAP (R58)
DRAQ5
Merged
control
1 day after PHx
2 days after PHx
4 days after PHx
14 days after PHx
25 µm

Fig. S11. Changes in transaminase concentrations and the activation of TGFb during liver regeneration in wt mice. (A) AST, ALT and LDH activity in the serum after PHx at the indicated time points (n=3-9). (B) Sections of snap-frozen liver tissue from wt control mice and after PHx at the indicated time points were stained with TGFb-LAP (green) and with DRAQ5 (blue). Representative sections are shown (Scale bar: 25 µm).

Fig. S12

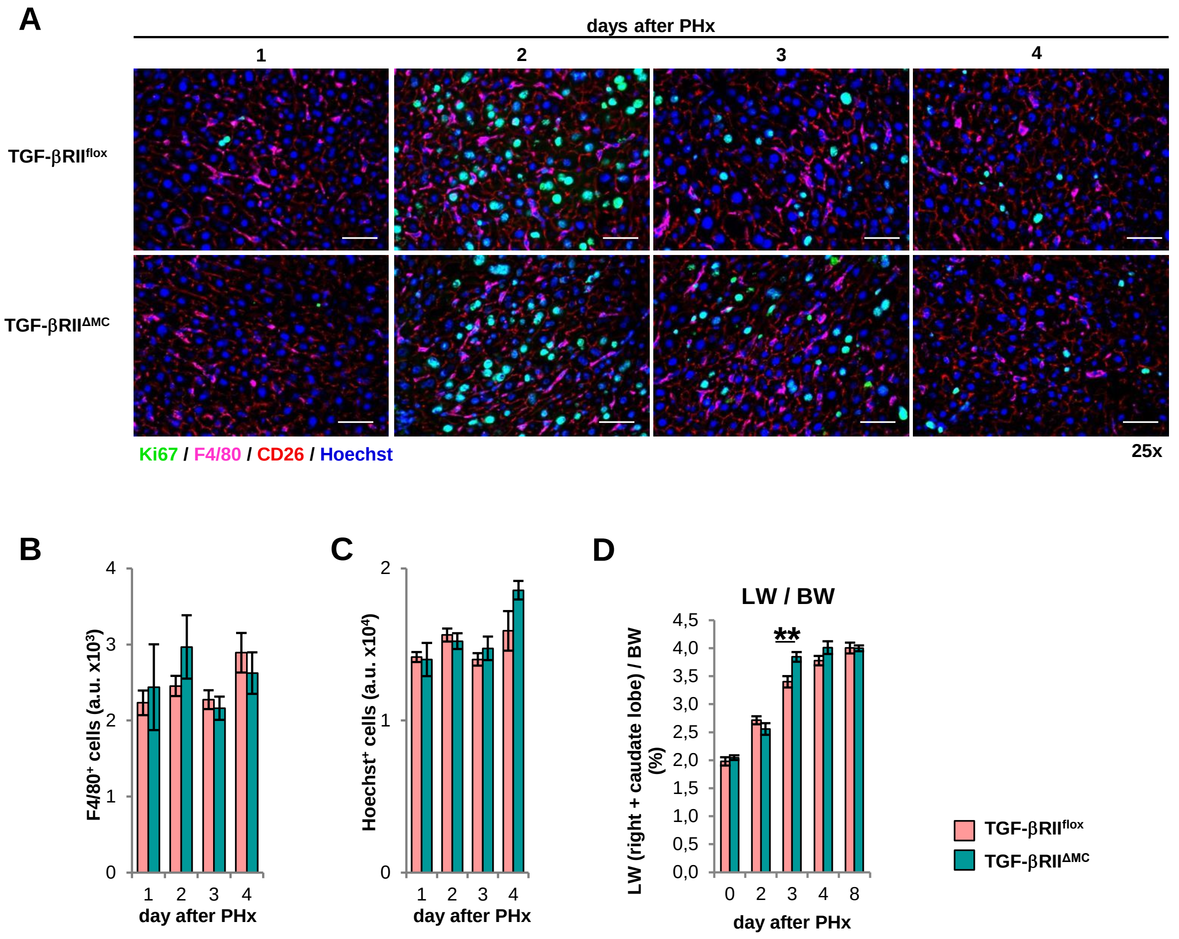

Fig. S12. Influence of the deletion of TGF-βRII in myeloid cells on the regenerating liver. (A) Sections of snap-frozen liver tissue from TGF-βRII^{flox} and TGF-βRII$^{\Delta MC}$ mice one to four days after PHx were stained with anti-Ki67 (green), anti-F4/80 (pink) and anti-CD26 (red) antibodies as well as with Hoechst (blue). Representative sections are shown (25x magnification, scale bars: 50 µm). (B) Quantification of macrophages (F4/80^{+} cells) and (C) cell nuclei (Hoechst^{+}) in liver sections (n=3-5). (D) Liver weight (LW, only of the right and caudate lobe) to body weight (BW) ratio was determined at the indicated time points in TGF-βRII^{flox} and TGF-βRII$^{\Delta MC}$ mice after PHx (n=5-8). The data are presented as means ± SEM. Significant differences are indicated by ** for $p \leq 0.01$ (Mann-Whitney U test).

Fig. S13

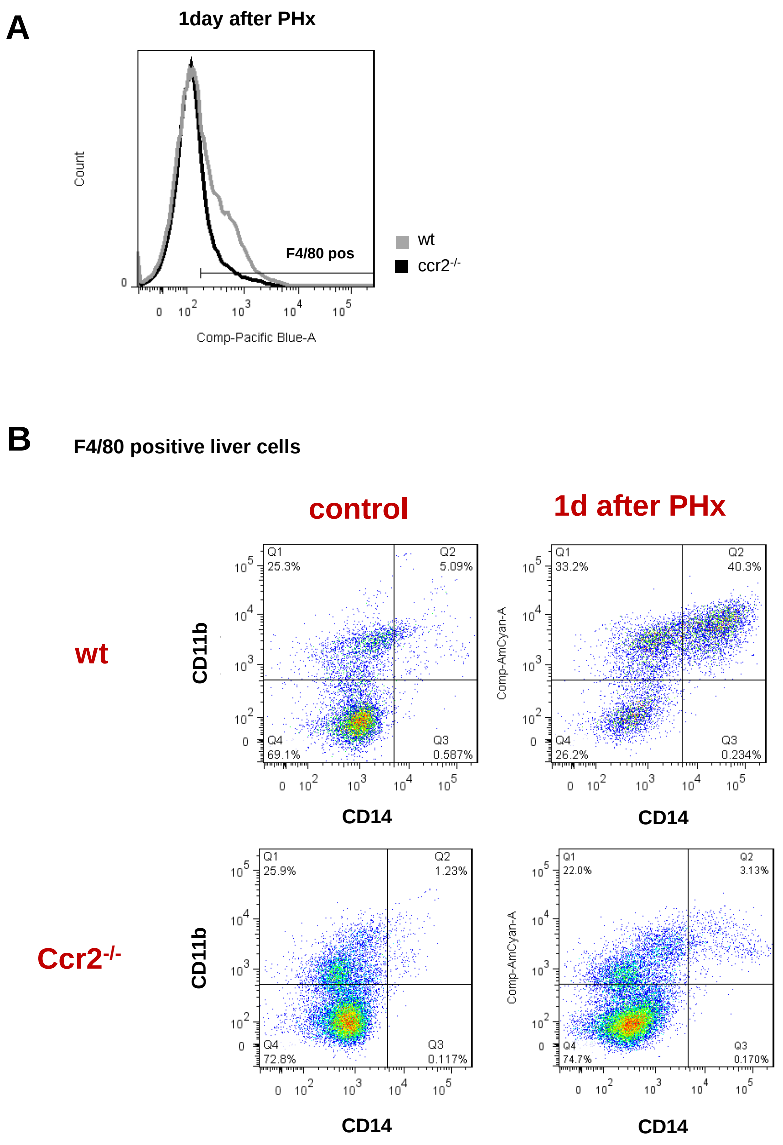

Fig. S13. Influence of CCR2 on the recruited macrophages after PHx. (A) Comparison of F4/80 positive liver cells from wt and CCR2$^{-/-}$ mice one day after PHx. (B) Analysis of the F4/80 positive cells in two parameter density plots for CD11b and CD14 in control animals as well as one day after PHx for wt and CCR2$^{-/-}$ animals.

Fig. S14

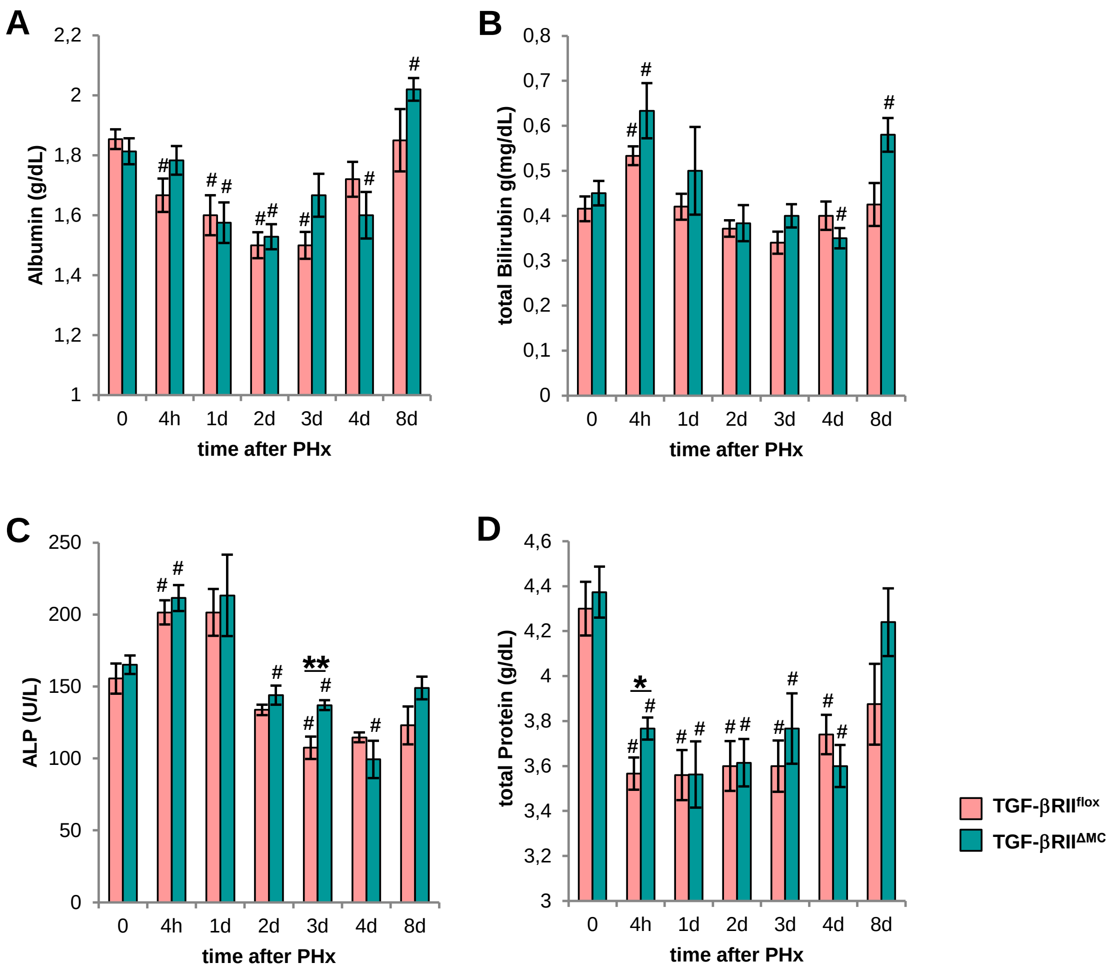

Fig. S14. Serum parameters of TGF-βRII^{flox} and TGF-βRII$^{\Delta MC}$ mice were compared after PHx. (A-D) Parameter activity or concentration was measured in serum from TGF-βRII^{flox} (gray) and TGF-βRII$^{\Delta MC}$ mice (black) after PHx at the indicated time points (n=4-8). Data are expressed as mean ± SEM. Significant differences from 0h are indicated by # for $p \leq 0.05$, and differences between TGF-βRII$^{\Delta MC}$ and TGF-βRII^{flox} mice are indicated by * for $p \leq 0.05$ and ** for $p \leq 0.01$ (Mann-Whitney U test).

Fig. S15

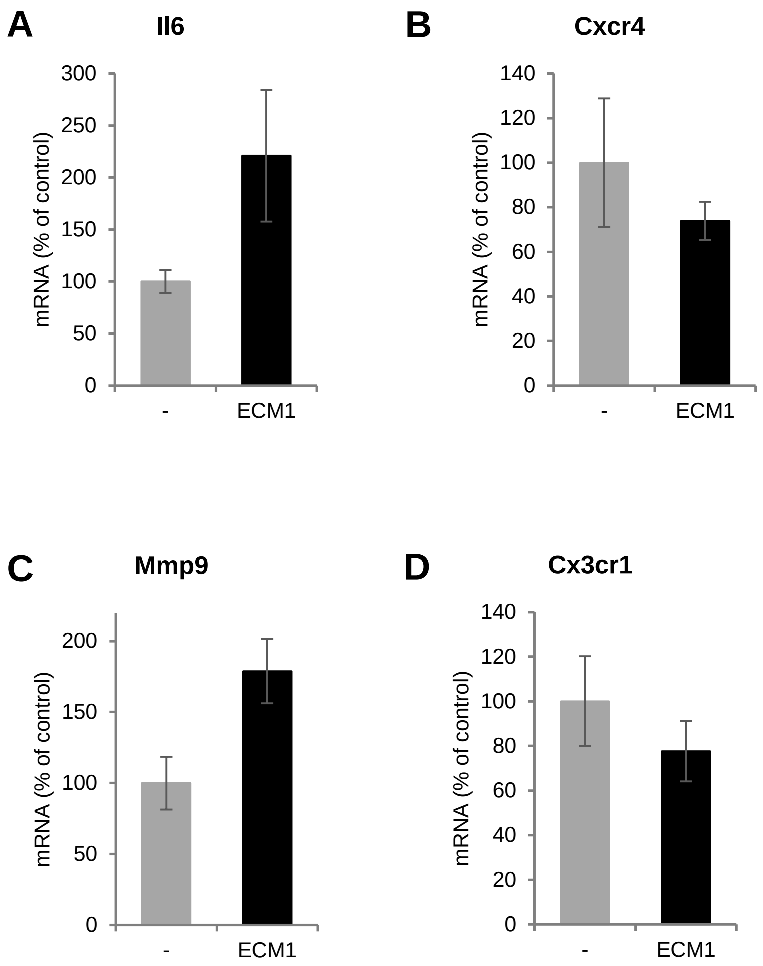

Fig. S15. The expression of IL-6, Cxcr4, Mmp9, and Cx3cr in BMDM tends to be modulated by ECM1 in a manner similar to the influence of hepatocytes. BMDM were generated from wt mice and stimulated for 24 hours with 25 ng/ml ECM1. Subsequently total RNA was prepared and expression of transcripts for IL-6, Mmp9, Cxcr4 and Cx3cr1 was analyzed by rtPCR using respective primers. Data are expressed as mean ± SEM (n=5).

Table S1: List of proteins and their lfq_log2 mean intensity that are unique in the respective proteome for the type of cultivation.

A unique in acrophages in co-culture

Gene name fasta	lfq_log2 mean intensity
METAP2	21,63
FYB1	22,84
NUDC	22,77
STK10	21,18
AIF1	22,46
EEF1B	22,78
ITGB5	24,48
NMT1	21,52
HCK	22,91
OAS1A	22,70
CD48	21,93
VAV1	23,06
C5AR1	24,73
SSB	22,95
PCYT1A	21,83
MCM5	22,02
ADPRH	21,96
RPL23	23,82
HNRNPH2	21,59
LILRB3	22,64
SIRPA	24,47
MOCOS	21,33
ARHGAP45	21,58
FXR1	22,07
NNT	22,36
TFRC	22,17
NDUFA12	22,07
PPP1R18	21,66
HAT1	21,88
EMC1	22,39
SMC2	22,02
STEAP3	23,34
GFM1	23,00
LRRC25	23,13
APBB1IP	22,68
HNRNPUL1	21,32
RPA1	23,04
YTHDF2	21,56
LPXN	22,85
BZW1	22,78
RER1	21,82
OCIAD1	22,80
SEC13	22,16
GATM	23,43
FMNL1	23,33
VAPA	23,07
LIPA	23,75

B unique in macrophages in monoculture

Gene name fasta	lfq_log2 mean intensity
SLC27A2	24,72
GPD1	24,81
VTN	22,51
VASP	23,83
GLS2	23,88
UGT1A1	21,94
BDH1	23,19
CES2E	23,67
UGT3A2	23,71
SUOX	22,89
UROC1	22,82
CES1F	24,94
BAAT	21,39
HIGD1A	22,06
SLC25A15	24,66

C unique in hepatocytes in co-culture

Gene name fasta	lfq_log2 mean intensity
GPD1L	20,97
CLIC4	23,99

D unique in hepatocytes in monoculture

Gene name fasta	lfq_log2 mean intensity
SQSTM1	21,11

Table S2: By hepatocytes induced significant changes in BMDM. The proteome of mono- and co-cultivated BMDM were analysed after two days of cultivation by mass spectrometry. All significant changed proteins are listed in the table with the respective average of 5 independent samples and the p-value (p≤0.5).

Gene_names	logFC	AveExpr	P.Value	adj.P.Val
CYP2F2	-4.665	22.258	0.000003	0.001
DMGDH	-4.654	24.285	0.001733	0.046
HMGCS2	-4.103	25.159	0.000280	0.019
PC	-3.940	24.365	0.000027	0.007
UOX	-3.921	23.031	0.001142	0.040
DECR2	-3.872	22.799	0.000002	0.001
OTC	-3.850	23.557	0.001791	0.046
SARDH	-3.816	23.798	0.002164	0.047
UGT2B17	-3.576	23.935	0.001869	0.046
ALDH4A1	-3.553	25.421	0.000248	0.019
CES3A	-3.433	24.478	0.005663	0.070
SLC25A22	-3.246	23.003	0.000057	0.011
KRT8	-3.210	23.731	0.008468	0.087
CPS1	-3.061	29.397	0.002228	0.047
EPHX2	-3.023	22.598	0.003385	0.059
MGST1	-2.819	26.167	0.010858	0.100
ALDH7A1	-2.716	25.046	0.008691	0.087
AASS	-2.626	22.698	0.013933	0.112
ASS1	-2.584	26.131	0.002145	0.047
RBP4	-2.568	24.244	0.023336	0.137
ALDH1L1	-2.464	22.199	0.006883	0.078
ISOC2A	-2.405	23.200	0.000155	0.016
EHHADH	-2.342	21.057	0.000453	0.023
NDUFA5	-2.305	24.035	0.000953	0.036
CA3	-2.302	24.123	0.033671	0.160
UQCRFS1	-2.285	25.137	0.001805	0.046
CLYBL	-2.231	23.965	0.000363	0.021
ADH1	-2.169	25.387	0.001830	0.046
ACOX1	-2.151	24.316	0.000276	0.019
LPL	-1.939	26.309	0.000464	0.023
AKR7A2	-1.721	22.828	0.005420	0.070
EPHX1	-1.716	21.769	0.000358	0.021
ATP5PB	-1.670	25.661	0.001202	0.040
DBT	-1.614	22.295	0.011526	0.103
ALDOC	-1.574	24.430	0.000924	0.036
SERPINE1	-1.555	22.214	0.025755	0.138
BHMT	-1.552	25.932	0.044873	0.179
SLC25A13	-1.494	25.291	0.003676	0.059
HSD17B10	-1.465	25.668	0.000618	0.026
LYPLA1	-1.425	22.391	0.001515	0.046
ECI1	-1.374	25.566	0.005061	0.067
BLVRB	-1.374	26.399	0.006216	0.075
HADH	-1.366	25.381	0.003213	0.058
GPX1	-1.325	22.917	0.013935	0.112
SELENBP2	-1.272	24.664	0.037100	0.165
PRDX6	-1.151	25.632	0.004922	0.067
DLD	-1.128	26.353	0.010876	0.100
GCDH	-1.025	23.130	0.027287	0.142
SFXN1	-1.021	22.856	0.013116	0.112
PRDX1	-1.004	31.117	0.013856	0.112
ACAA2	-1.002	27.471	0.030475	0.152

Gene_names	logFC	AveExpr	P.Value	adj.P.Val
SRRT	1,011	21,888	0,034654	0,161
ASPH	1,018	24,200	0,035981	0,164
SEPT7	1,032	24,076	0,033162	0,160
GANAB	1,090	25,968	0,028628	0,145
DDX39B	1,092	25,092	0,008470	0,087
CTSC	1,111	25,077	0,002745	0,052
RRBP1	1,127	25,502	0,015036	0,113
RPSA	1,132	26,829	0,004134	0,062
ERP44	1,137	24,863	0,003521	0,059
SDCBP	1,142	23,036	0,002151	0,047
FDPS	1,152	23,816	0,015526	0,115
TRIM28	1,158	24,141	0,022297	0,137
TUBA1B	1,167	28,811	0,034254	0,161
SEPT2	1,180	24,688	0,010205	0,097
SRSF3	1,181	25,915	0,028987	0,146
MCM2	1,191	23,739	0,013668	0,112
RPL12	1,193	25,789	0,006868	0,078
NSF	1,193	24,054	0,025776	0,138
UGGT1	1,196	24,826	0,024819	0,138
DNAJB11	1,198	24,265	0,007577	0,082
RARS	1,207	23,501	0,024966	0,138
AP1B1	1,213	22,981	0,025883	0,138
UBA52	1,232	26,829	0,004406	0,062
RPS27L	1,232	25,308	0,027731	0,143
CD36	1,235	25,147	0,021030	0,136
USP5	1,241	23,451	0,046827	0,183
CMPK1	1,255	23,705	0,004637	0,064
ANPEP	1,259	25,025	0,008078	0,086
ITGA5	1,260	23,384	0,025446	0,138
OXCT1	1,273	25,357	0,025461	0,138
ATIC	1,277	24,888	0,047335	0,183
GNS	1,281	24,102	0,006852	0,078
RPL38	1,287	23,356	0,005681	0,070
MCM7	1,297	24,019	0,004189	0,062
PLA2G15	1,300	23,888	0,018072	0,128
MYO1C	1,307	23,780	0,023335	0,137
SLC25A4	1,313	23,699	0,000129	0,016
NUDT21	1,318	23,239	0,031435	0,155
SNX2	1,336	25,721	0,039843	0,172
PLXNB2	1,357	25,292	0,003148	0,058
STOM	1,363	25,039	0,030884	0,153
EHD1	1,363	24,906	0,015086	0,113
PCK2	1,365	24,394	0,009796	0,094
FTL1	1,399	25,101	0,001500	0,046
ADSS	1,401	24,262	0,008693	0,087
PPP2CA	1,450	24,367	0,034878	0,161
SPTAN1	1,484	24,656	0,007066	0,079
VWA5A	1,545	24,906	0,031854	0,156
CANX	1,547	25,259	0,001194	0,040
PSAP	1,584	24,823	0,002655	0,052
LRPPRC	1,600	24,251	0,008582	0,087
ITGA6	1,616	24,083	0,000535	0,025
SRSF7	1,674	25,241	0,034588	0,161
HEXB	1,687	27,040	0,047025	0,183
ESYT1	1,740	24,588	0,007601	0,082
DPP3	1,756	22,994	0,014418	0,112
GLB1	1,774	24,253	0,003667	0,059
SAMM50	1,791	22,510	0,002530	0,051
FCGR1	1,849	23,529	0,000167	0,016
DDX3X	1,944	24,510	0,004258	0,062
ARPC3	2,001	25,715	0,014303	0,112
ATP2B1	2,010	24,165	0,001953	0,046
AP2A2	2,031	24,089	0,003990	0,062
CTSS	2,067	25,411	0,014531	0,112
SLC3A2	2,170	24,430	0,022487	0,137
PRMT1	2,182	23,604	0,000091	0,014
MPEG1	2,219	23,610	0,001756	0,046
ACTC1	2,249	26,634	0,009624	0,093
HEXA	2,269	25,647	0,023414	0,137
NAP1L1	2,577	24,002	0,000562	0,025
TLN1	2,730	27,022	0,002544	0,051

Table S3: Antibodies for flow cytometry

A for NPLC

Name	Fluorochrome	Supplier	Cat no.	Clone no.
F4/80	eFluor450	Thermo Fischer	48-4801-82	BM8
CD11b	Brilliant Violet 510	BioLegend	101245	M1/70
CD14	PE	Thermo Fischer	12-0141-81	Sa2-8
CD163	PerCP-eFluor 710	Thermo Fischer	46-1631-82	TNKUPJ
CD169	PE-Cy7	BioLegend	142412	3D6.112
CD206	Alexa Flour 647	BioLegend	141711	C068C2
MHCII	FITC	Thermo Fischer	11-5321-85	M5/114.15.2
CCR2	FITC	R&D systems	FAB5538F	475301
Cx3cr1	PE-Cy7	BioLegend	149015	SA0011F11

B for BMDM

Primary Antibodys

Name	Fluorochrome	Supplier	Cat no.	Clone no.
CD11b	PE	Thermo Fischer	12-0112-81	M1/70
CD14	PE	Thermo Fischer	12-0141-81	Sa2-8
CD163	-	Dianova	T-2123	2F8
CD206	FITC	Bio-Rad	MCA2235FB	MR5D3
CXCR4 (CD184)	PE	Thermo Fischer	12-9991-81	2B11
F4/80	APC	Thermo Fischer	17-4801-80	BM8
MHCII	PE	Thermo Fischer	12-5321-82	M5/114.15.2
CD169	-	Bio-Rad	MCA884	3D6.112

Secondary Antibodys and Isotype Controls

Name	Fluorochrome	Supplier	Cat no.	Clone no.
goat@rat	Cy3	Dianova	112-165-044	polyclonal
Rat IgG2b kappa Isotype Control	PE	Thermo Fischer	12-4031-81	eB149/10H5
Rat IgG2b kappa Isotype Control	APC	Thermo Fischer	17-4031-81	eB149/10H5
Rat IgG2a kappa Isotype Control	FITC	Thermo Fischer	11-4321-81	eBR2a

Table S4: Antibodies for immunofluorescence staining

Name	Supplier	Cat no.	Clone no.
TGF-b-LAP	Cosmo Bio	CAC-RIK-MA-R58	18F9-16
Ki67	Abcam	ab15580	SP6
F4/80	Bio Rad	MCA497RT	A3-1
CD26	R&D Systems	AF954-SP	polyclonal
AlexaFluor 488 AffiniPure F8 (ab')2 Donkey Anti-Mouse IgG (H+L)	Jackson ImmunoResearch	715-546-151	polyclonal
Donkey IgG anti-Rabbit IgG (Fc)-FITC	Dianova	SEC-183422	polyclonal
Donkey IgG anti-Rat IgG (H+L)-Cy5	Dianova	712-175-150	polyclonal
Donkey IgG anti-Goat IgG (H+L)-Cy3	Dianova	705-165-147	polyclonal

Table S5: Sequences of qRT-PCR oligonucleotide primers Supplier: Eurofins genomics (Ebersberg, Germany), all primers are generated for mouse samples

gene name	sequence (forward)	sequence (reverse)
a2M	5'-CCTCGGCTGAGGTAGAGATG-3'	5'-TGAGCCACTTCACAATGAGC-3'
ApoE	5'-AACCGCTTCTGGGATTACCT-3'	5'-ATCAGTGCCGTCAGTTCTTG-3'
Arg1	5'-GTGAAGAACCCACGGTCTGT-3'	5'-CTGGTTGTCAGGGGAGTGTT-3'
Ccl2	5'-GGCTGGAGAGCTACAAGAGG-'3	5'-ATGTCTGGACCCATTCCTTC-3'
Ccl3	5'-ACCATGACACTCTGCAACCA-3'	5'-GATGAATTGGCGTGGAATCT-3'
Ccl4	5'-GCCCTCTCTCTCCTCTTGCT-3'	5'-CCGGGAGGTGTAAGAGAAAC-3'
CD11c	5'-CTGGATAGCCTTTCTTCTGCTG -3'	5'-GCACACTGTGTCCGAACTC-3'
CD163	5'-TGCTGGATCTCCTGGTTGTA-3'	5'-TCCAGGAGCGTTAGTGACAG-3'
Siglec1 (CD169)	5´-GACCAGCTTGTAGCCTCCTC-3´	5´-AGCAGCTATAGAGCCCCTCA-3´
Comp	5'-AACCCAGACCAGCGTAACTC-3'	5'-GGCCATCCAGGTCTGTATCT-3'
Crp	5'-GGGTGGTGCTGAAGTACGAT-3'	5'-GGTTTCCCATCAATCCAGAA-3'
Cxcl1	5'-ACCCAAACCGAAGTCATAGC-3'	5'-TGGGGACACCTTTTAGCATC-3'
Cxcl2	5'-GCCAAGGGTTGACTTCAAGA-3'	5'-GCCCTTGAGAGTGGCTATGA-3'
Cxcl3	5'-AACACCCTACCAAGGGTTGA-3'	5'-GGGTTGAGGCAAACTTCTTG-3'
Cx3cr1	5´-GCACGGTGTCACCATTAGTC-3´	5´-AGTCACCCAGACACTCGTTG-3´
Cxcr4	5'-CCTAGCTTTCTTTGCCTGCT-3'	5'-TCTCGAAGTCACATCCTTGC-3'
CD32 (Fcgr2)	5'-TCCATGTGTTCTCACGGACT-3'	5'-GACCACAGCCTTTGGAAGAT-3'
CD16 (Fcgr3)	5'-CACCATCACTGTCCAAGACC-3'	5'-GTGTCCACTGCAAACAGGAG-3'
Fizz1	5'-GTCCTGGAACCTTTCCTGAG-3'	5'-CAGGGAGATGCAGATGAGAA-3'
Hgf	5'-GATTGGATCAGGACCATGTG-3'	5'-CCAGGAACAATGACACCAAG-3'
Hprt	5'-TCCTCCTCAGACCGCTTTT-3'	5'-CCTGGTTCATCATCGCTAATC-3'
Icam1	5'-CGCTGTGCTTTGAGAACTGT-3'	5'-CAGAGGTCTCAGCTCCACACT-3'
IFNb	5'-CCCTATGGAGATGACGGAGA-3'	5'-ACCCAGTGCTGGAGAAATTG-3'
IL-10	5'-CCAAGCCTTATCGGAAATGA-3'	5'-TCCTGAGGGTCTTCAGCTTC-3'
IL-12p35	5'-GACCAAACCAGCACATTGAA-3'	5'-AGCTCCCTCTTGTTGTGGAA-3'
IL-1b	5'-TCACAGCAGCACATCAACAA-3'	5'-TGTCCTCATCCTGGAAGGTC-3'
IL-6	5'-GTTGCCTTCTTGGGACTGAT-3'	5'-CAGGTCTGTTGGGAGTGGTA-3'
Irf4	5´-ACCGAGATTCCAGGTGACTC-3´	5´-GTCTGGCTAGCAGAGGTTCC-3´
Mmp9	5´-TCTTCTGGCGTGTGAGTTTC-3´	5´-ACTGCAGGAGGTCGTAGGTC-3´
CD206 (Mrc1)	5'-CAGGTGTGGGCTCAGGTAGT-3'	5'-TGTGGTGAGCTGAAAGGTGA-3'
Nfil3	5´-GCAGGTGACGAACATTCAAG-3´	5´-CCACCACACCTGTTTTGAAG-3´
Nos2 (iNos)	5'-AGAGATTGGAGGCCTTGTGT-3'	5'-ACATGCAAGGAAGGGAACTC-3'
Osm	5'-AAGCCACAGCTGCCTATCTT-3'	5'-GAGATACCTGAGCCCACACA-3'
PD-L1 (CD274)	5'-ACTTGCTACGGGCGTTTACT-3'	5'-TCCCGTTCTACAGGGAATCT-3'
PD-L2 (PdcD1lg2)	5'-TCCACATCCCTAGTGTCCAA-3'	5'-GCTTTGACTTTCACCGTCAG-3'
Ppp2r1a	5'-ACCTGGAGGCCTTAGTGATG-3'	5'-CTGCTTTCTGGAGCTCTGTG-3'
Pten	5'-ACACCGCCAAATTTAACTGC-3'	5'-TACACCAGTCCGTCCCTTTC-3'
Sbno2	5´-AGAACGCCCTGTTCCAATAC-3´	5´-AGGTCTAGGATGCCCATGTC-3´
Sdha	5'-TGGGGAGTGCCGTGGTGTCA-3'	5'-GTGCCGTCCCCTGTGCTGGT-3'
Smad3	5´-AGTGACCACCAGATGAACCA-3´	5´-TGACTGGCTGTAGGTCCAAG-3´
Socs3	5´-GCTCCAAAAGCGAGTACCAGC-3´	5´-AGTAGAATCCGCTCTCCTGCAG-3´
Sphk1	5'-CAACTGGCCTACCTTCCTGT-3'	5'-CCTCCAGAGGAACAAGGTGT-3'
Stab1	5'-CCCTCCTTCTGCTCTGTGTC-3'	5'-CAAACTTGGTGTGGATGTCG-3'
TGFβi	5'-AGCTCTTCCGCTCTCAAATC-3'	5'-TCTCAGGCCTCAGCTTTTCT-3'
Thsp1	5´-AGTGCAAAGACGTCGATGAG-3´	5´-GGCAGTTGTAGCCAGGATCT-3´
Tlr4	5'-AGAGCCGGAAGGTTATTGTG-3'	5'-CTGCTCAGAAACTGCCATGT-3'
Tnfsf14	5'-ACCCAGCAGCACATCTTACA-3'	5'-CCCATCATGATACGTCAAGC-3'
Tnfα	5'-GCTGAGCTCAAACCCTGGTA-3'	5'-CGGACTCCGCAAAGTCTAAG-3'
Ym1	5'-TCTGAATGAAGGAGCCACTG-3'	5'-AGCCACTGAGCCTTCAACTT-3'

References specific for supplementary materials

[1] Levéen P, Larsson J, Ehinger M, Cilio CM, Sundler M, Sjöstrand LJ, et al. Induced disruption of the transforming growth factor beta type II receptor gene in mice causes a lethal inflammatory disorder that is transplantable. Blood 2002;100:560-568.

[2] Boring L, Gosling J, Chensue SW, Kunkel SL, Farese RV, Jr., Broxmeyer HE, et al. Impaired monocyte migration and reduced type 1 (Th1) cytokine responses in C-C chemokine receptor 2 knockout mice. J Clin Invest 1997;100:2552-2561.

[3] Godoy P, Hengstler JG, Ilkavets I, Meyer C, Bachmann A, Müller A, et al. Extracellular matrix modulates sensitivity of hepatocytes to fibroblastoid dedifferentiation and transforming growth factor beta-induced apoptosis. Hepatology 2009;49:2031-2043.

[4] Rex J, Albrecht U, Ehlting C, Thomas M, Zanger UM, Sawodny O, et al. Model-Based Characterization of Inflammatory Gene Expression Patterns of Activated Macrophages. PLoS computational biology 2016;12:e1005018.

[5] Mitchell C, Willenbring H. A reproducible and well-tolerated method for 2/3 partial hepatectomy in mice. Nature protocols 2008;3:1167-1170.

[6] Poschmann G, Seyfarth K, Besong Agbo D, Klafki HW, Rozman J, Wurst W, et al. High-fat diet induced isoform changes of the Parkinson's disease protein DJ-1. Journal of proteome research 2014;13:2339-2351.

[7] Heming S, Hansen P, Vlasov A, Schwörer F, Schaumann S, Frolovaitė P, et al. MSPypeline: A python package for streamlined data analysis of mass spectrometry-based proteomics. Bioinformatics Advances 2022.

[8] Vizcaino JA, Csordas A, Del-Toro N, Dianes JA, Griss J, Lavidas I, et al. 2016 update of the PRIDE database and its related tools. Nucleic acids research 2016;44:11033.

[9] Bolstad BM, Irizarry RA, Astrand M, Speed TP. A comparison of normalization methods for high density oligonucleotide array data based on variance and bias. Bioinformatics (Oxford, England) 2003;19:185-193.

[10] Hao Y, Hao S, Andersen-Nissen E, Mauck WM, 3rd, Zheng S, Butler A, et al. Integrated analysis of multimodal single-cell data. Cell 2021;184:3573-3587.e3529.

[11] Stuart T, Butler A, Hoffman P, Hafemeister C, Papalexi E, Mauck WM, 3rd, et al. Comprehensive Integration of Single-Cell Data. Cell 2019;177:1888-1902.e1821.

[12] Butler A, Hoffman P, Smibert P, Papalexi E, Satija R. Integrating single-cell transcriptomic data across different conditions, technologies, and species. Nature biotechnology 2018;36:411-420.

[13] McGinnis CS, Murrow LM, Gartner ZJ. DoubletFinder: Doublet Detection in Single-Cell RNA Sequencing Data Using Artificial Nearest Neighbors. Cell systems 2019;8:329-337.e324.

[14] Korsunsky I, Millard N, Fan J, Slowikowski K, Zhang F, Wei K, et al. Fast, sensitive and accurate integration of single-cell data with Harmony. Nature methods 2019;16:1289-1296.

[15] Aizarani N, Saviano A, Sagar, Mailly L, Durand S, Herman JS, et al. A human liver cell atlas reveals heterogeneity and epithelial progenitors. Nature 2019;572:199-204.
